# Supplementary figures and images for: H3K9me1/2 methylation limits the lifespan of daf-2 mutants in C. elegans
Source: eLife. 2022 Sep 20;11:e74812. doi: 10.7554/eLife.74812 (PMC9514849; doi:10.7554/eLife.74812)

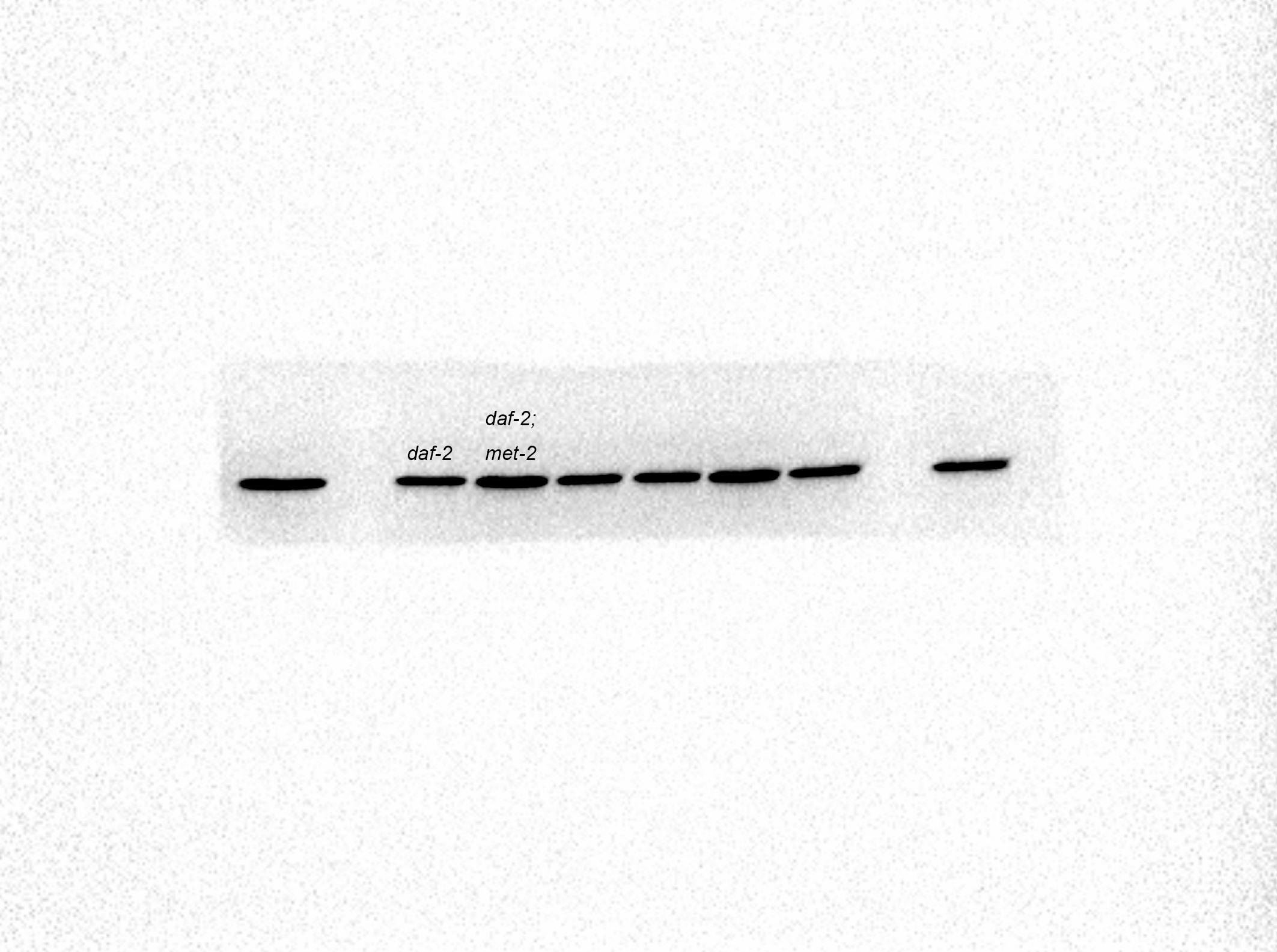

Supplement: Figure 2—source data 1. [file elife-74812-fig2-data1.zip › source data 1/figure2A/Actin-met-2.tif]

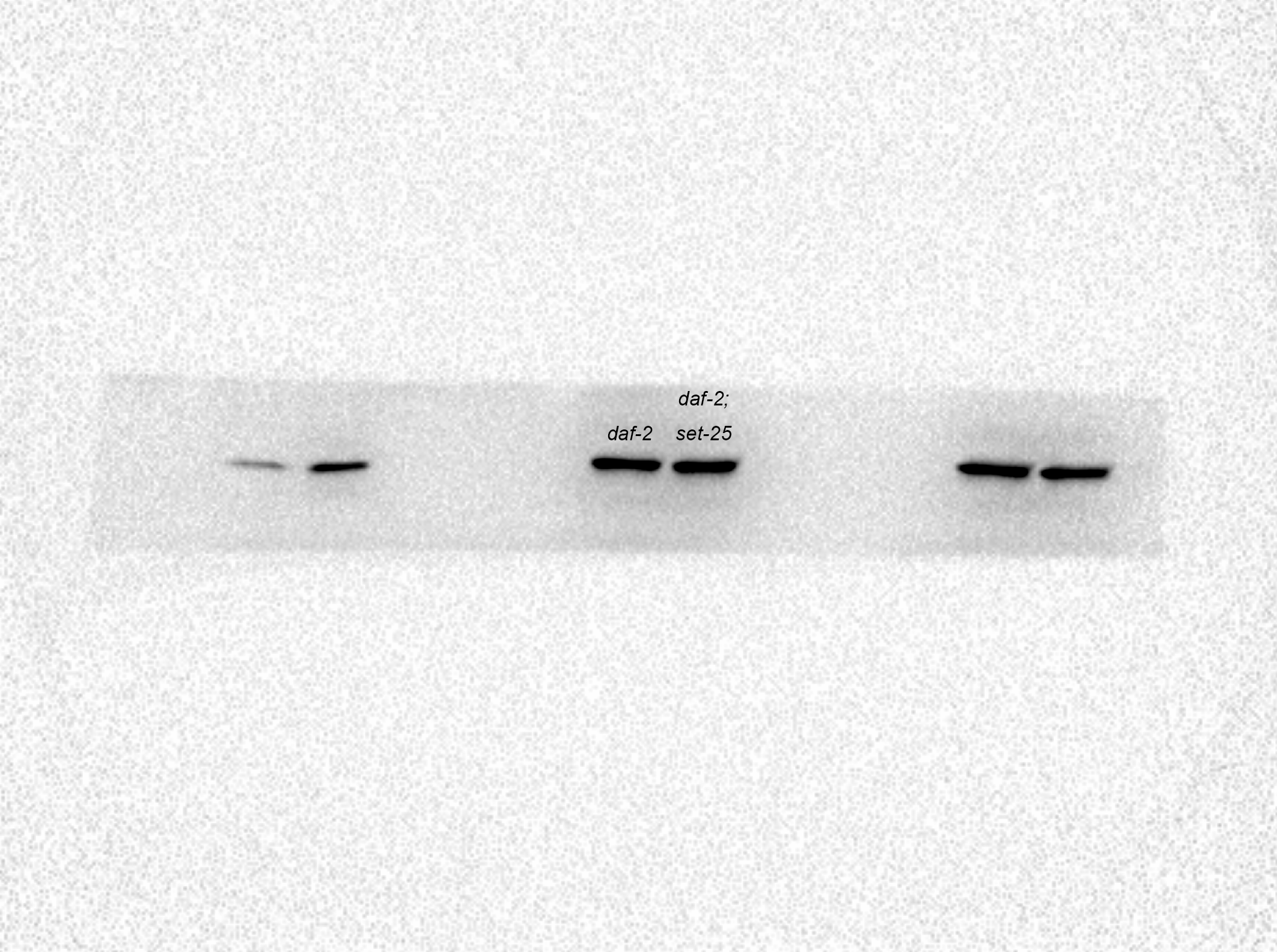

Supplement: Figure 2—source data 1. [file elife-74812-fig2-data1.zip › source data 1/figure2A/Actin-set-25.tif]

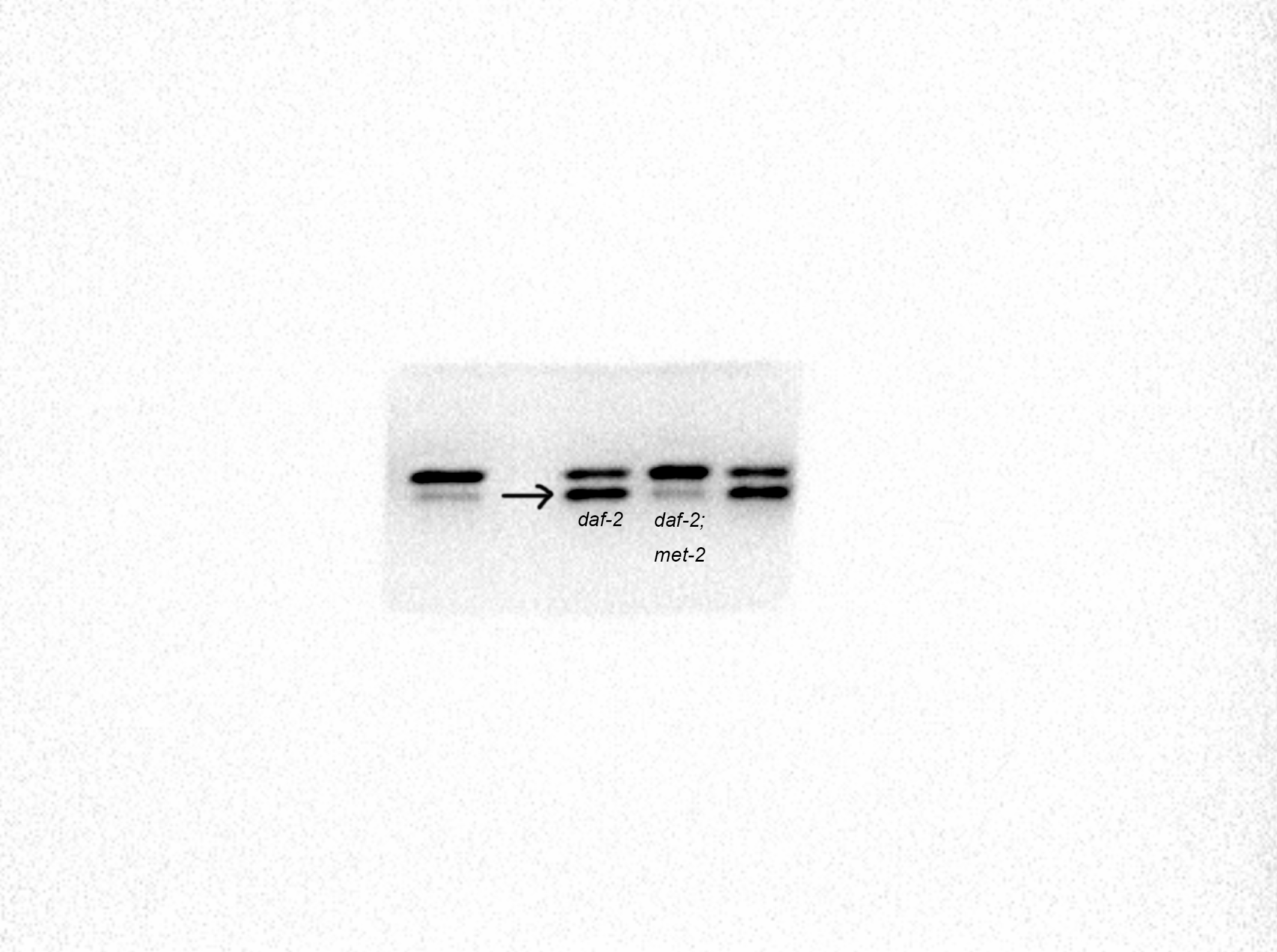

Supplement: Figure 2—source data 1. [file elife-74812-fig2-data1.zip › source data 1/figure2A/H3K9me1-met-2.tif]

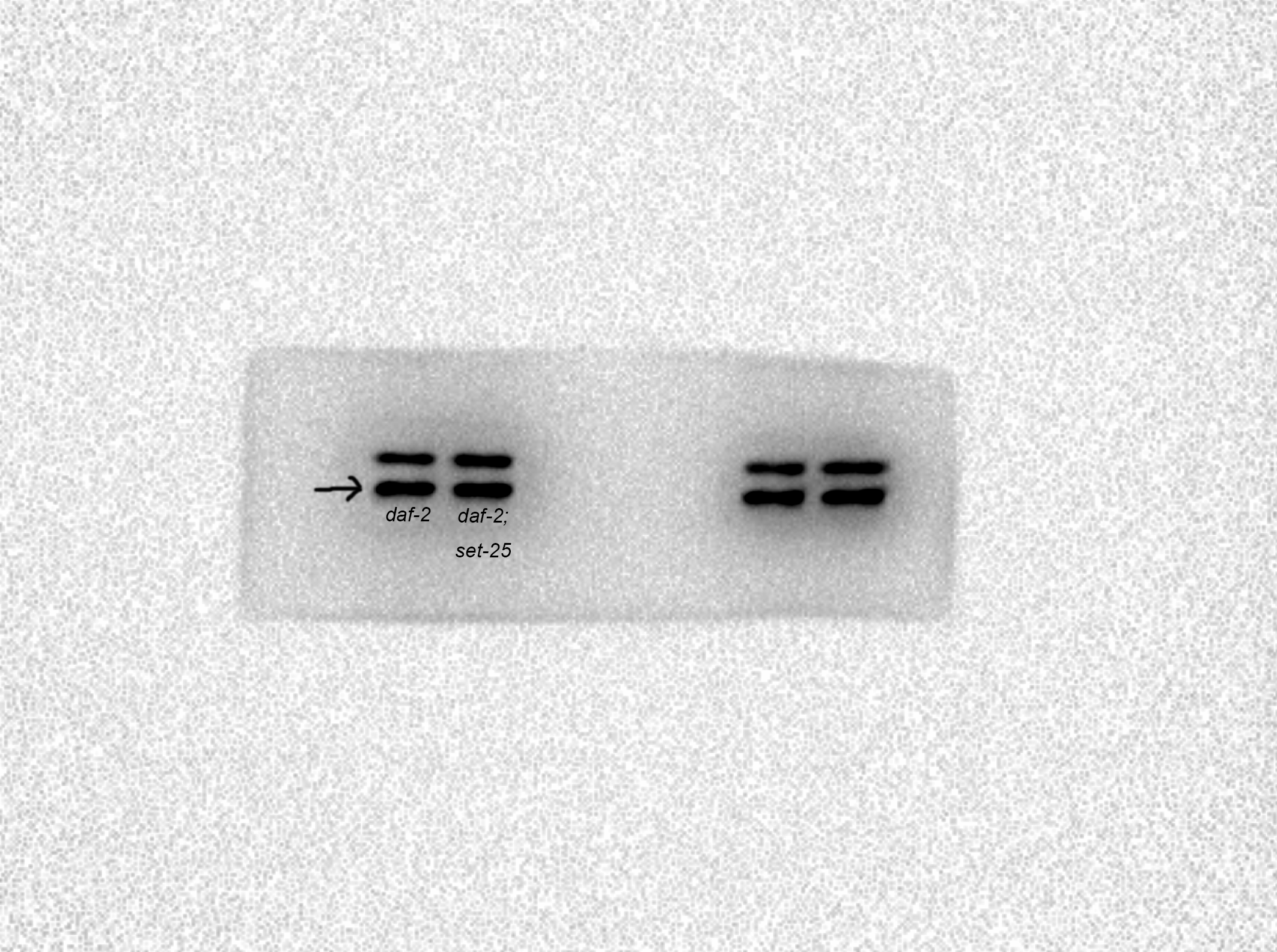

Supplement: Figure 2—source data 1. [file elife-74812-fig2-data1.zip › source data 1/figure2A/H3K9me1-set-25.tif]

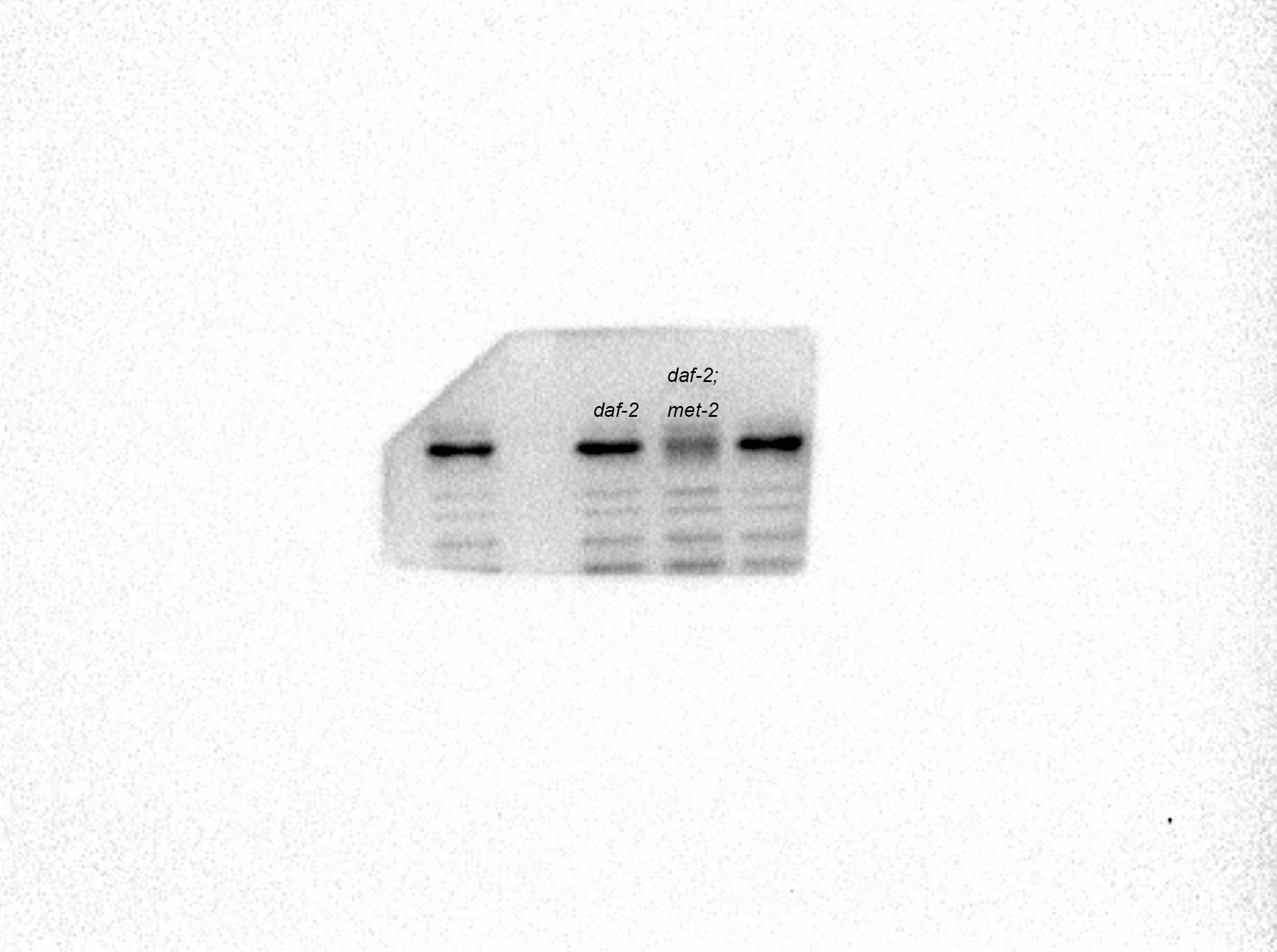

Supplement: Figure 2—source data 1. [file elife-74812-fig2-data1.zip › source data 1/figure2A/H3K9me2-met-2.tif]

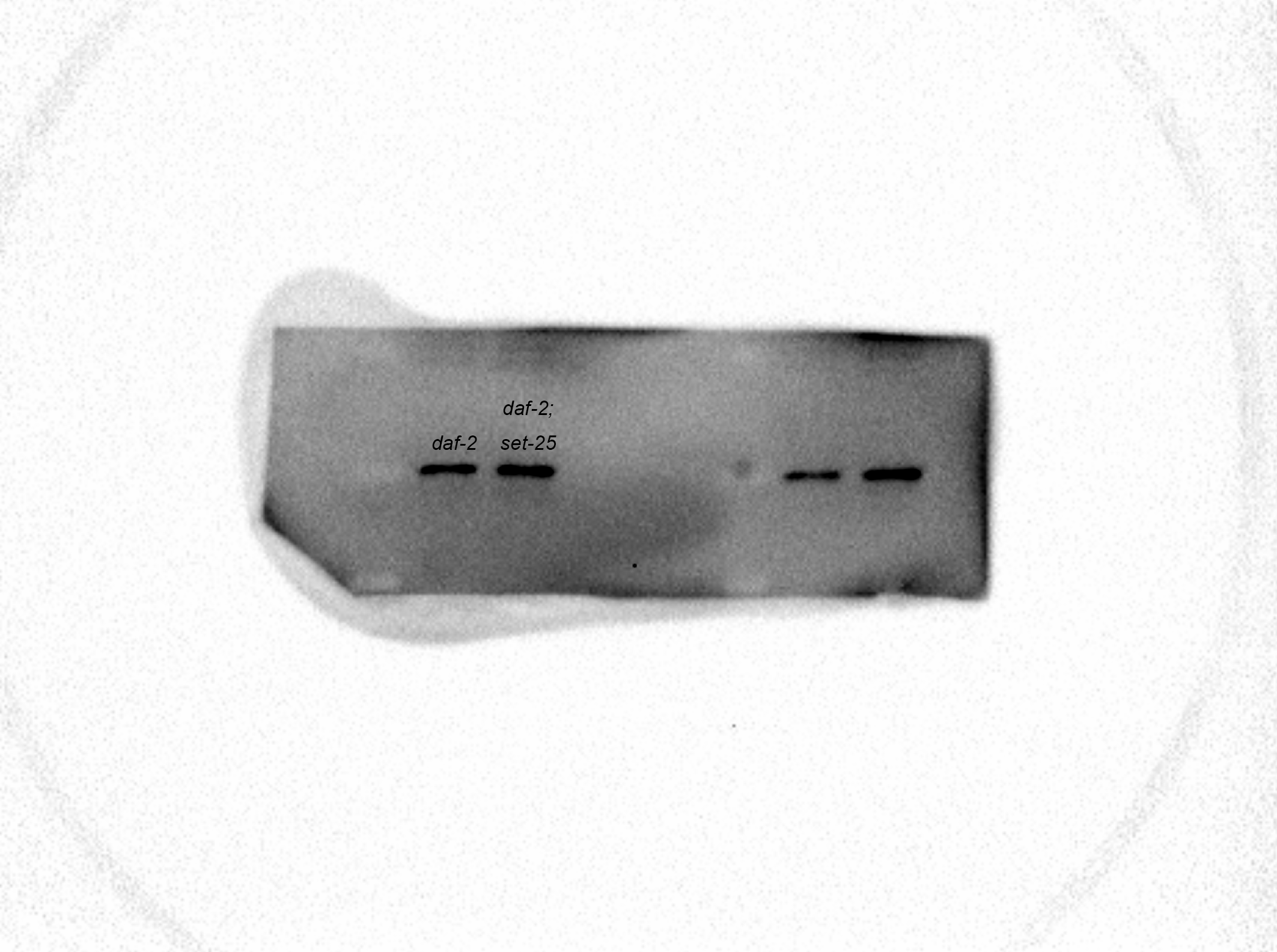

Supplement: Figure 2—source data 1. [file elife-74812-fig2-data1.zip › source data 1/figure2A/H3K9me2-set-25.tif]

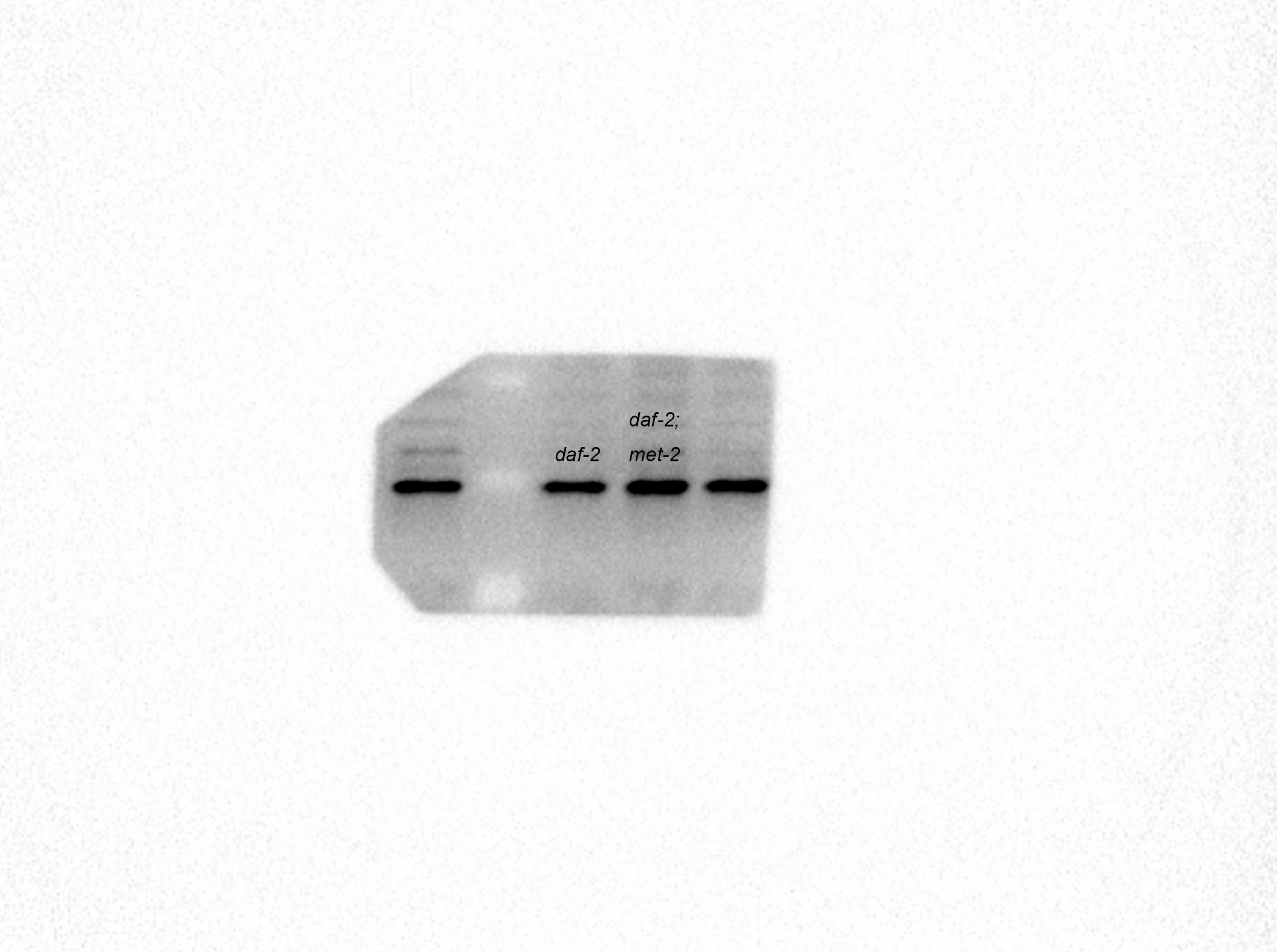

Supplement: Figure 2—source data 1. [file elife-74812-fig2-data1.zip › source data 1/figure2A/H3K9me3-met-2.tif]

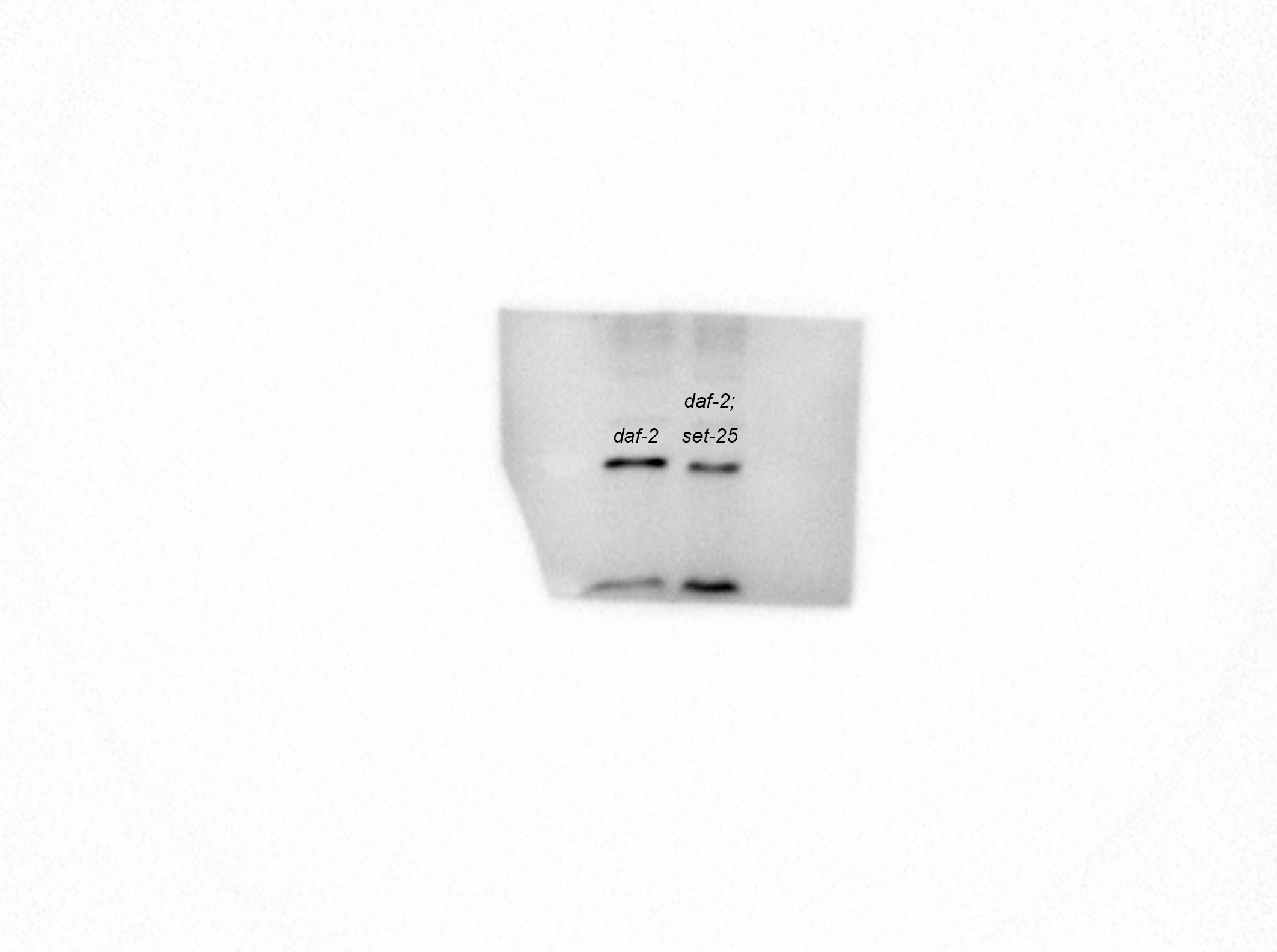

Supplement: Figure 2—source data 1. [file elife-74812-fig2-data1.zip › source data 1/figure2A/H3K9me3-set-25.tif]

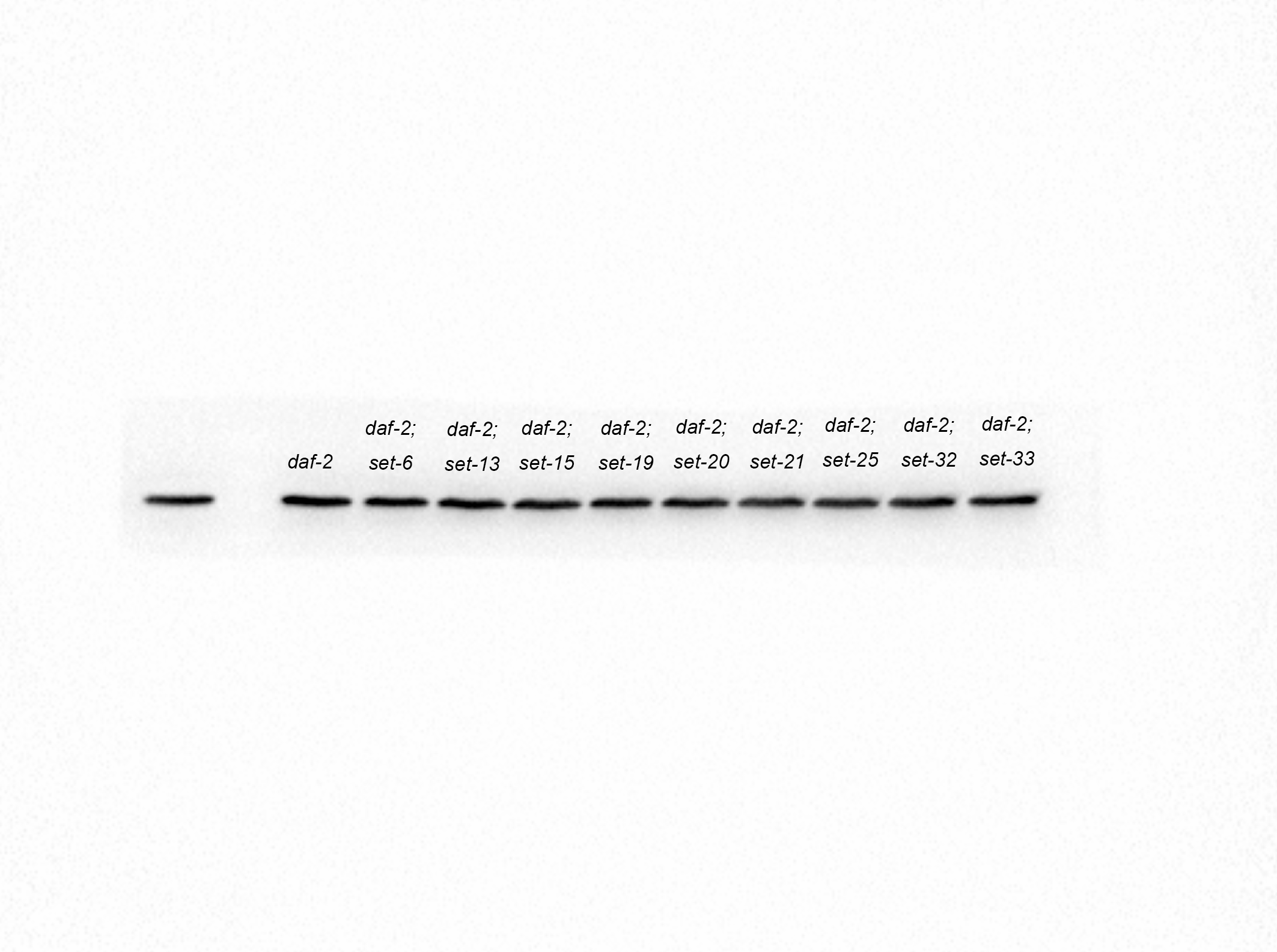

Supplement: Figure 8—source data 1. [file elife-74812-fig8-data1.zip › source data 2/figure8A/Actin.tif]

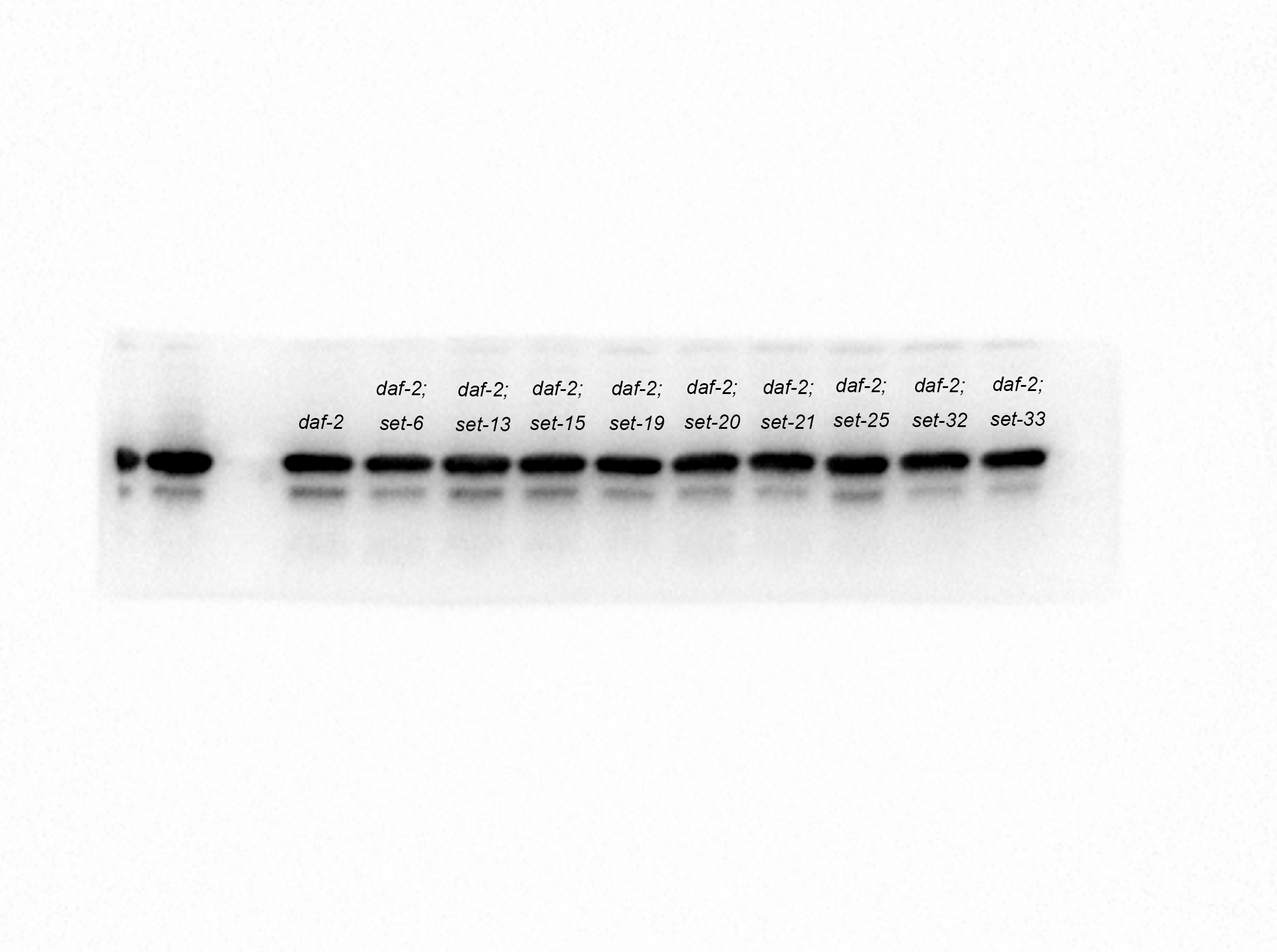

Supplement: Figure 8—source data 1. [file elife-74812-fig8-data1.zip › source data 2/figure8A/H3.tif]

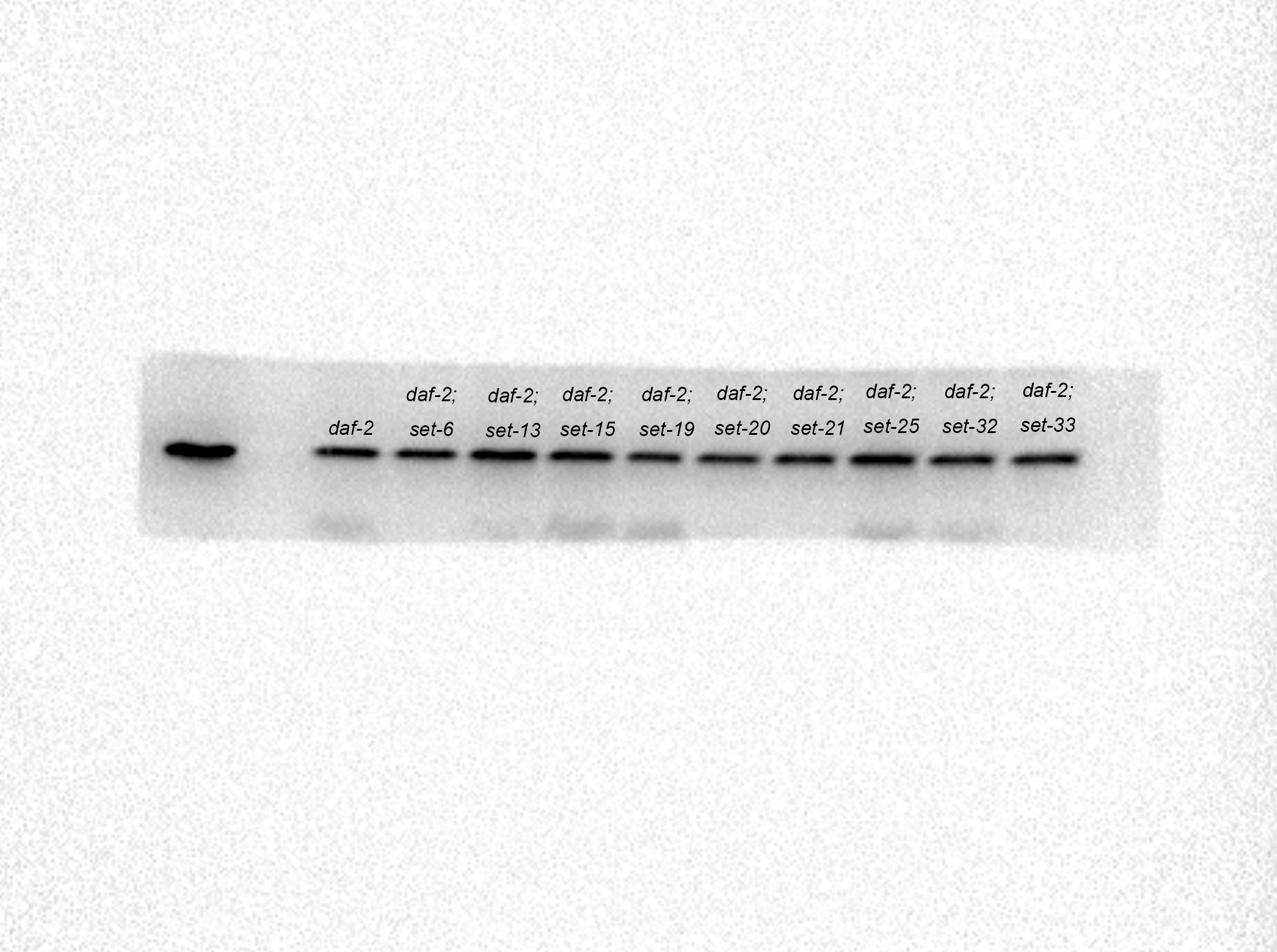

Supplement: Figure 8—source data 1. [file elife-74812-fig8-data1.zip › source data 2/figure8A/H3K23me2.tif]

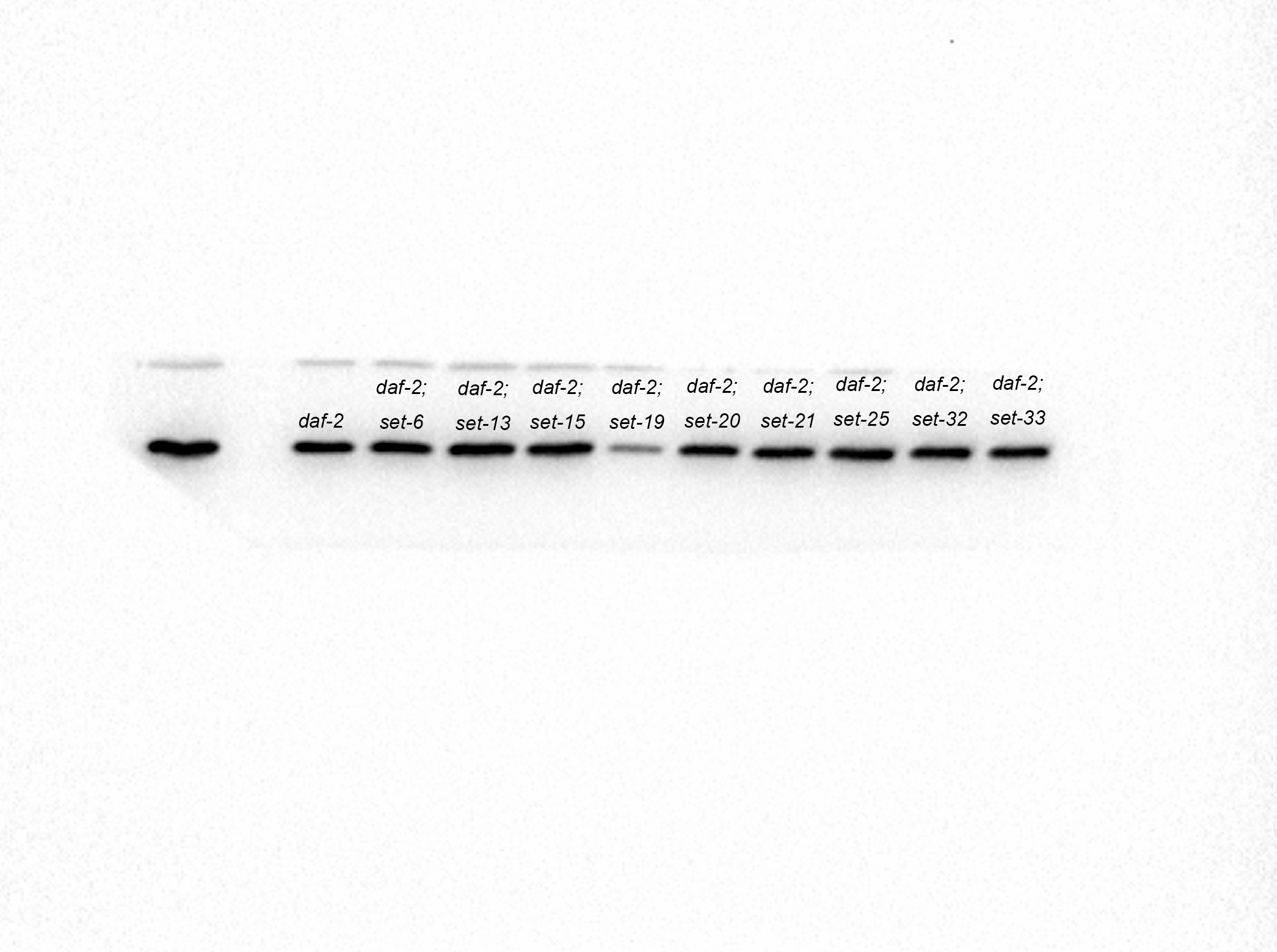

Supplement: Figure 8—source data 1. [file elife-74812-fig8-data1.zip › source data 2/figure8A/H3K23me3.tif]

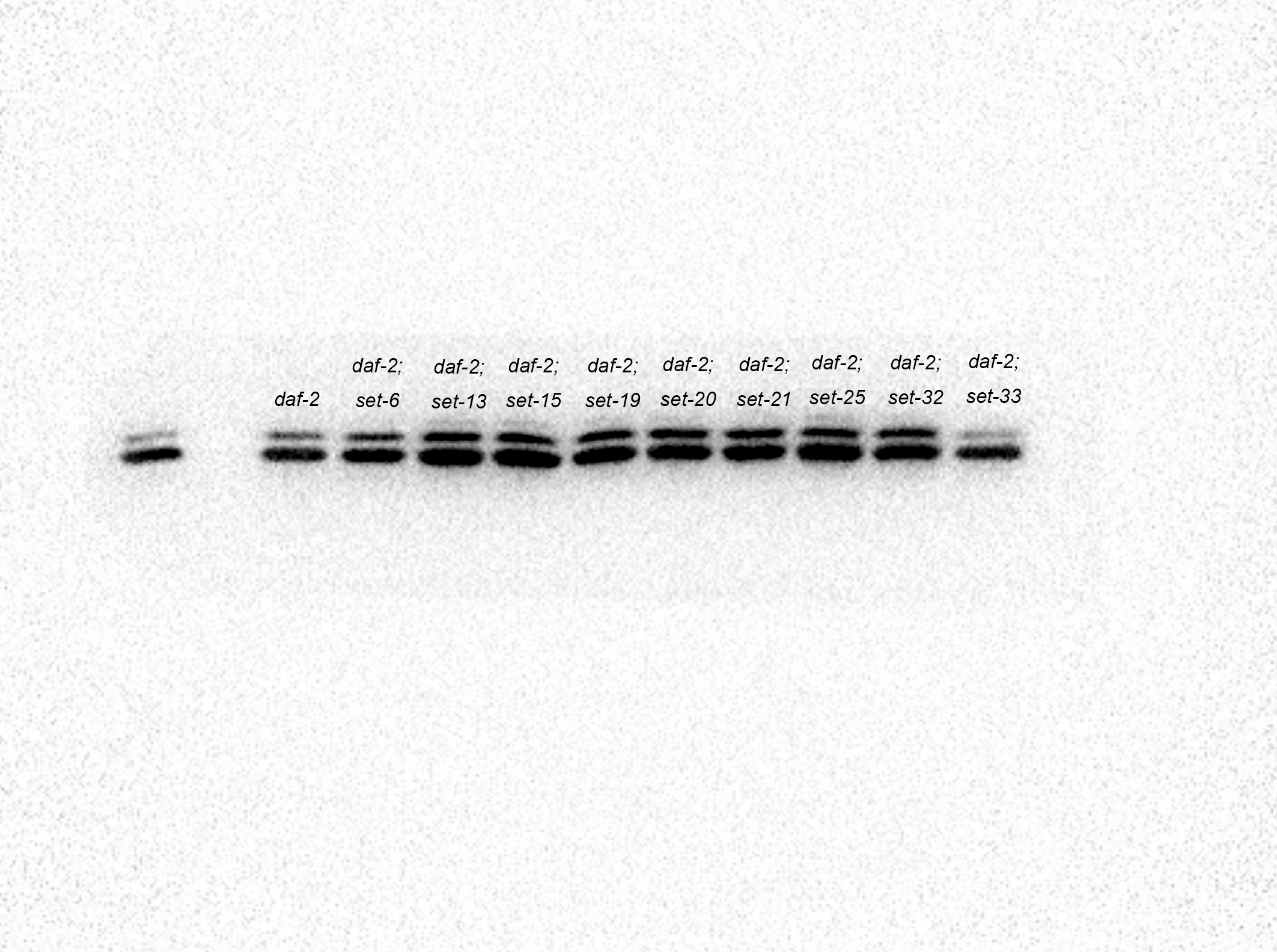

Supplement: Figure 8—source data 1. [file elife-74812-fig8-data1.zip › source data 2/figure8A/H3K27me1.tif]

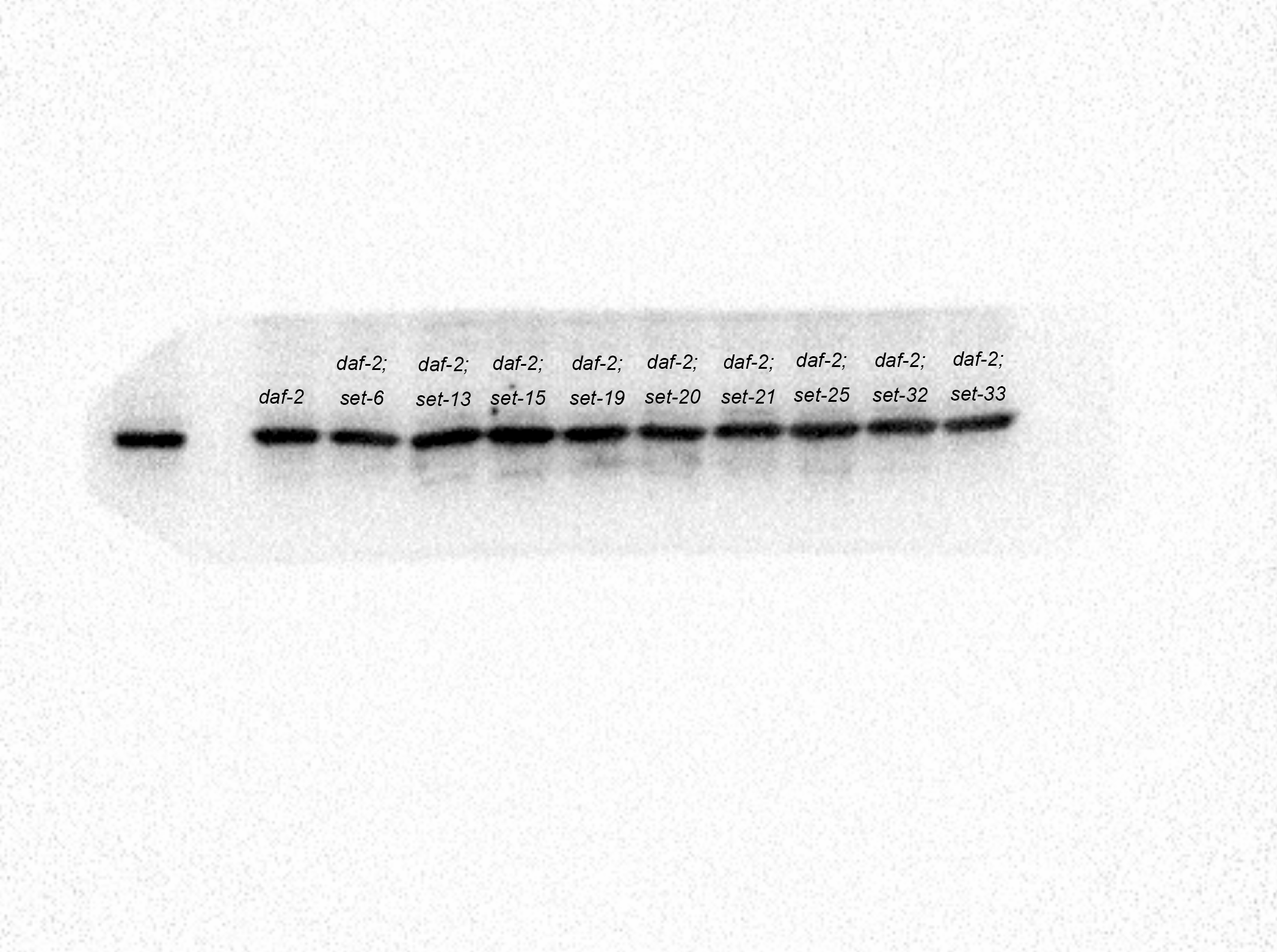

Supplement: Figure 8—source data 1. [file elife-74812-fig8-data1.zip › source data 2/figure8A/H3K27me2.tif]

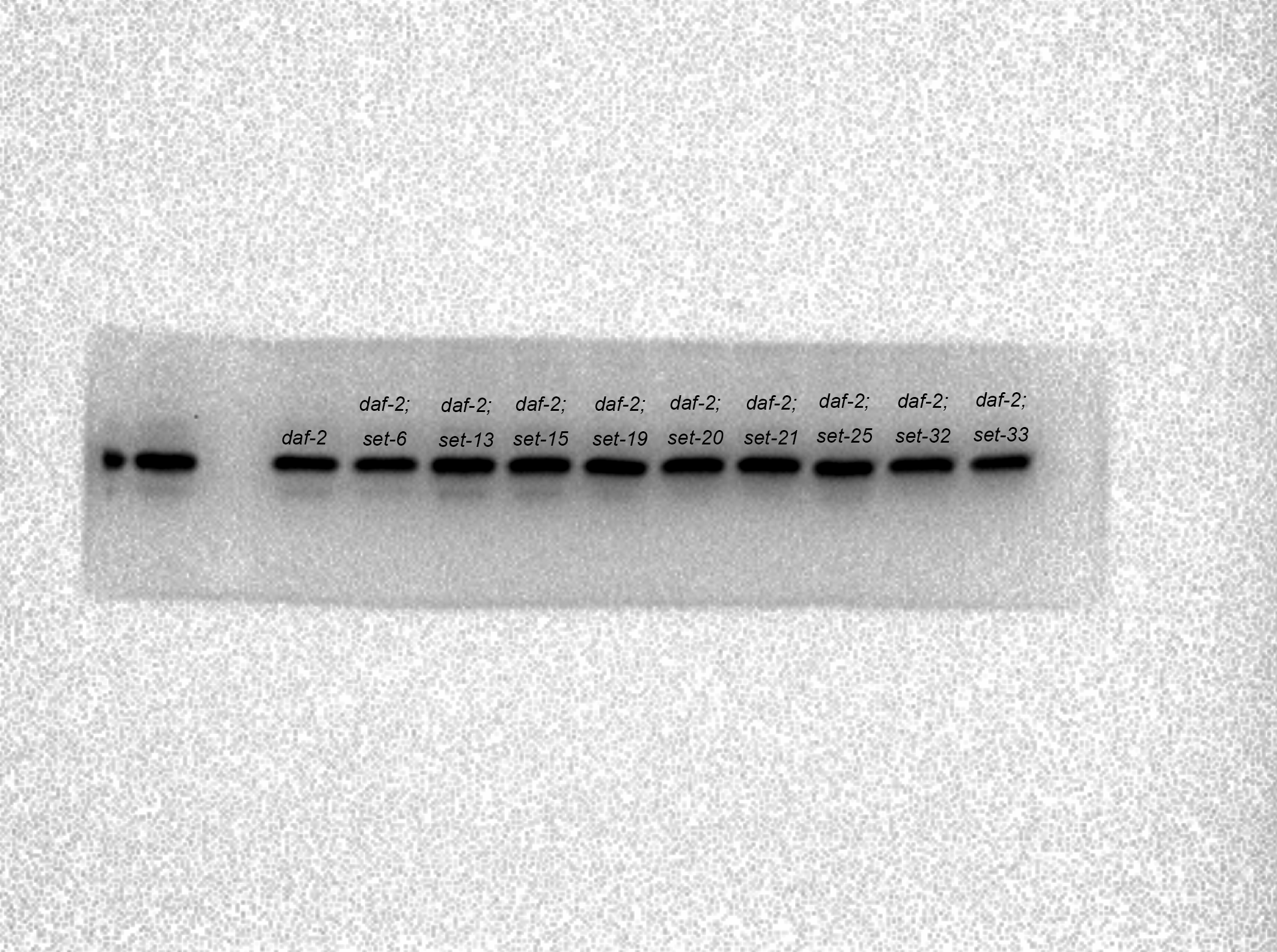

Supplement: Figure 8—source data 1. [file elife-74812-fig8-data1.zip › source data 2/figure8A/H3K27me3.tif]

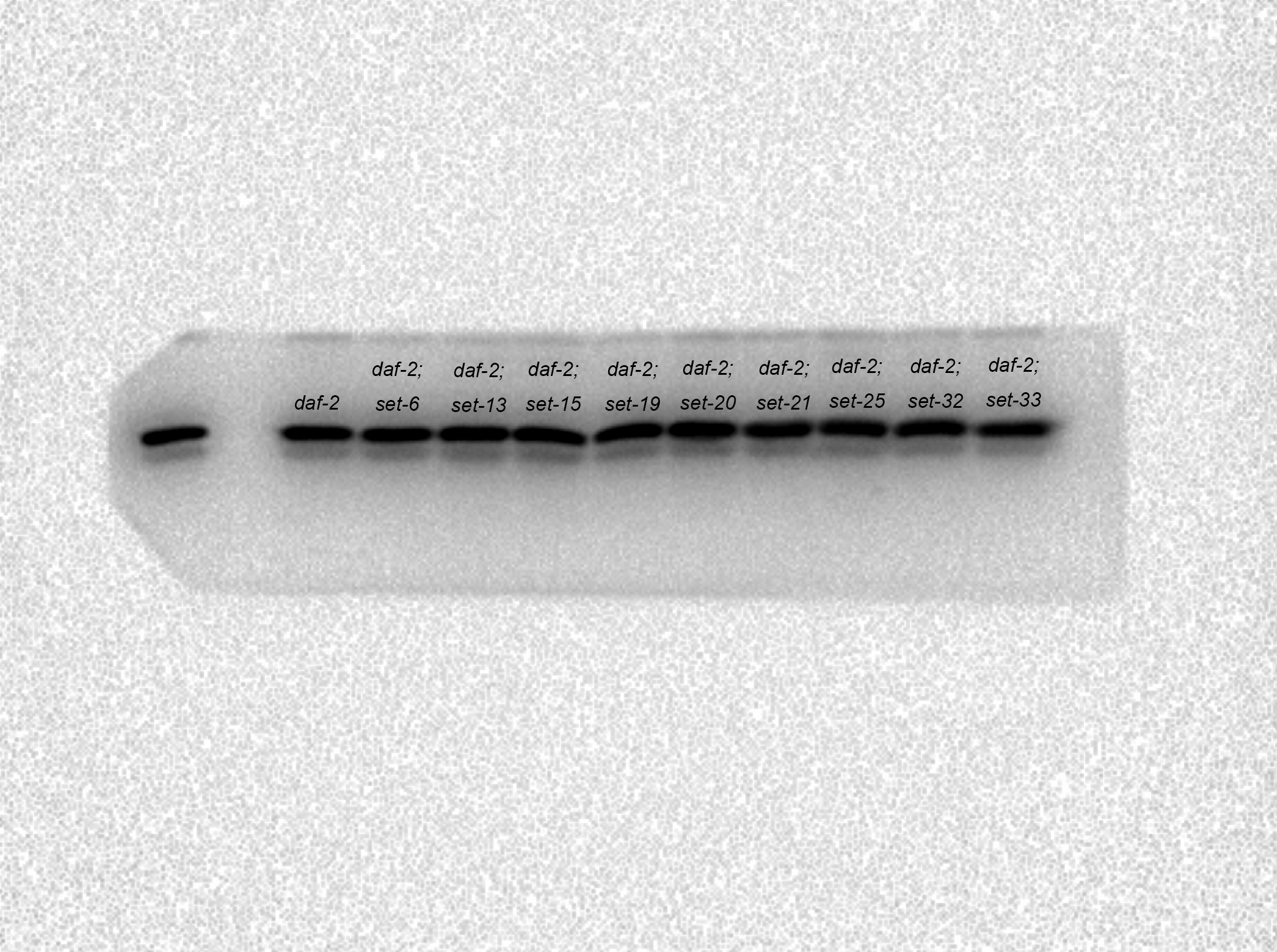

Supplement: Figure 8—source data 1. [file elife-74812-fig8-data1.zip › source data 2/figure8A/H3K36me1.tif]

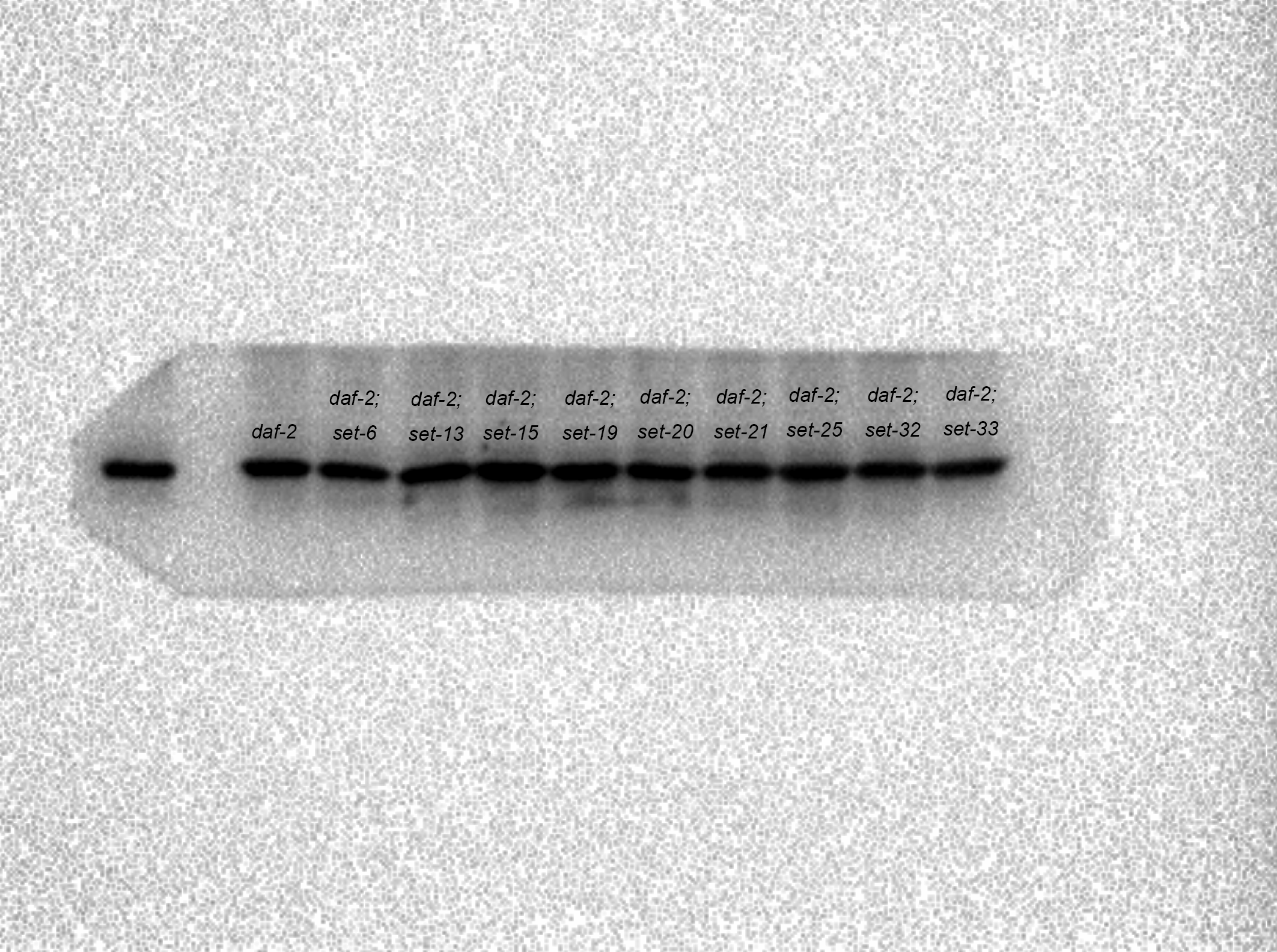

Supplement: Figure 8—source data 1. [file elife-74812-fig8-data1.zip › source data 2/figure8A/H3K36me2.tif]

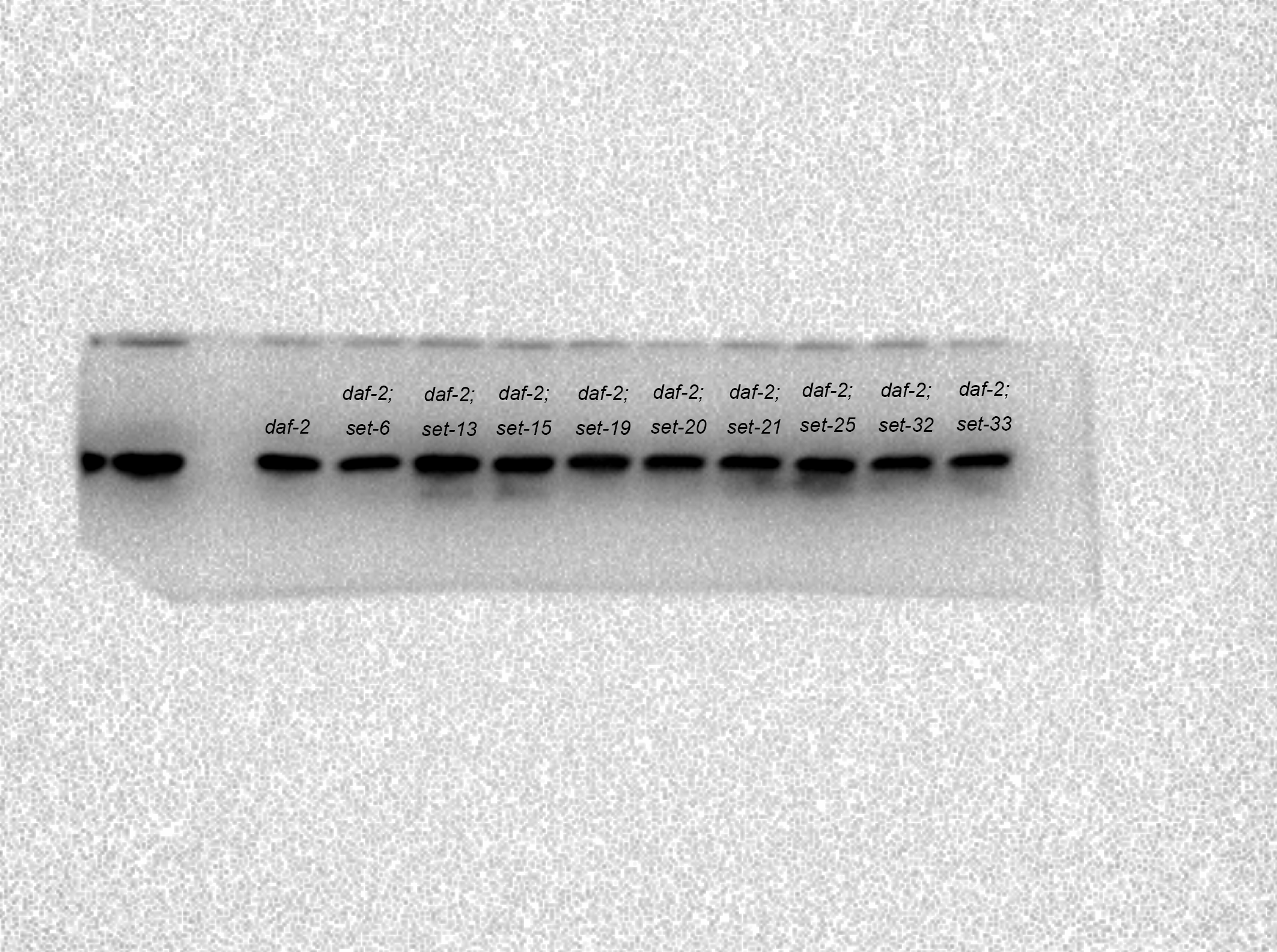

Supplement: Figure 8—source data 1. [file elife-74812-fig8-data1.zip › source data 2/figure8A/H3K36me3.tif]

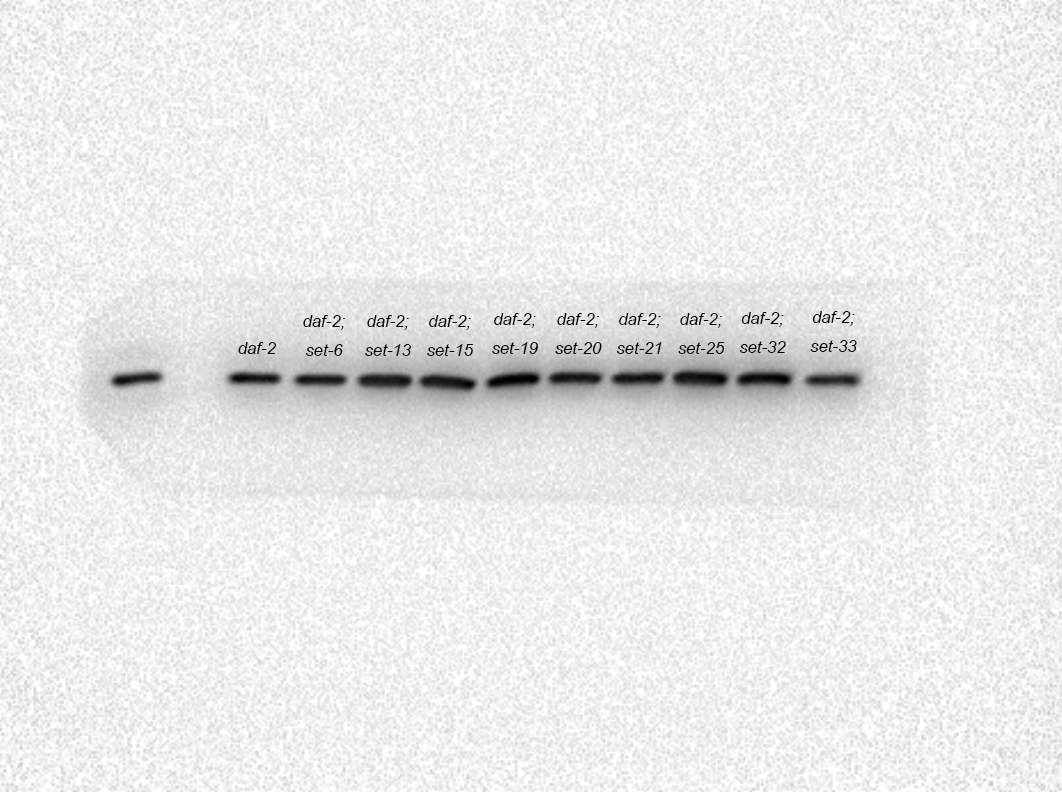

Supplement: Figure 8—source data 1. [file elife-74812-fig8-data1.zip › source data 2/figure8A/H3K4me1.tif]

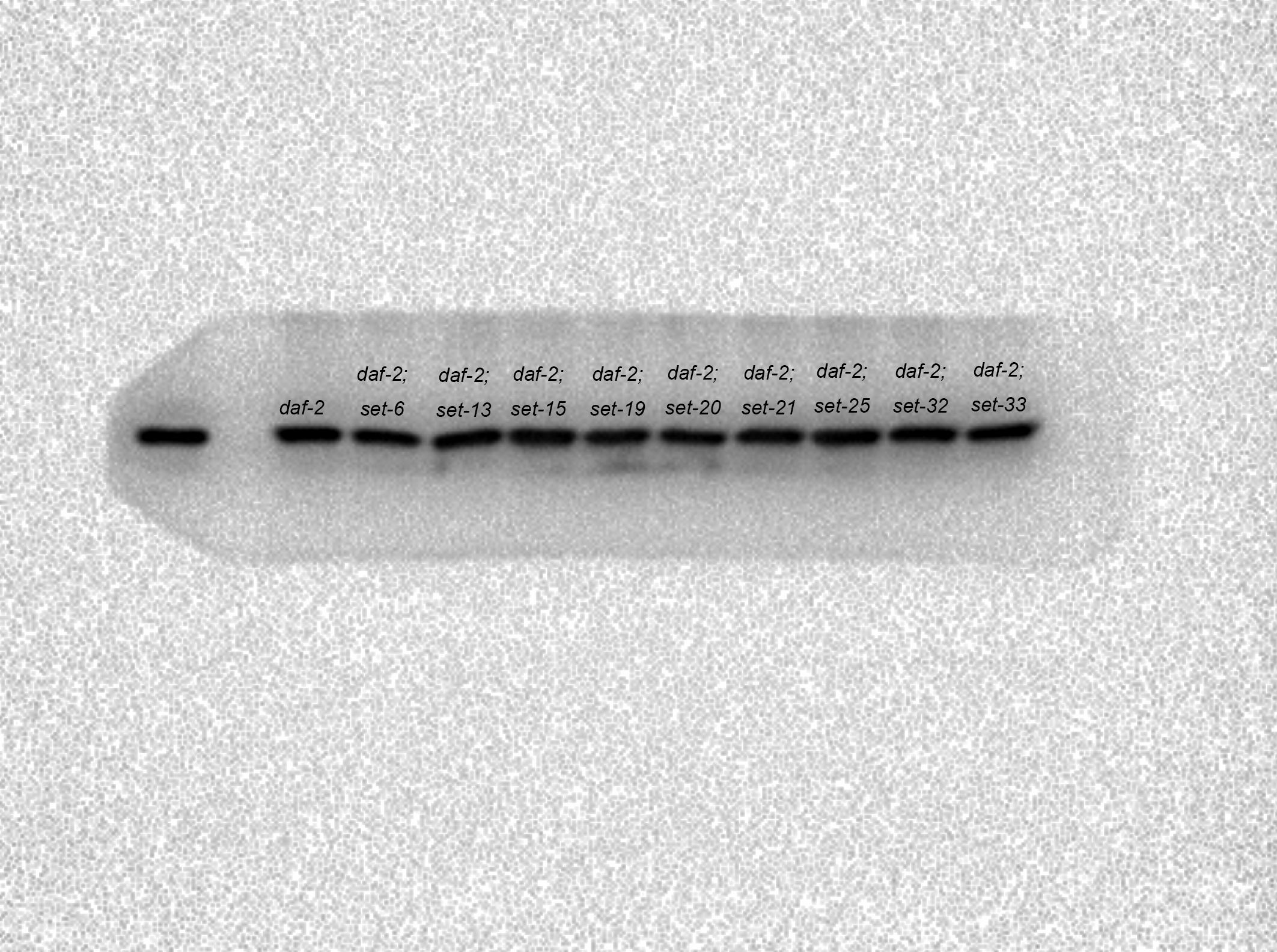

Supplement: Figure 8—source data 1. [file elife-74812-fig8-data1.zip › source data 2/figure8A/H3K4me2.tif]

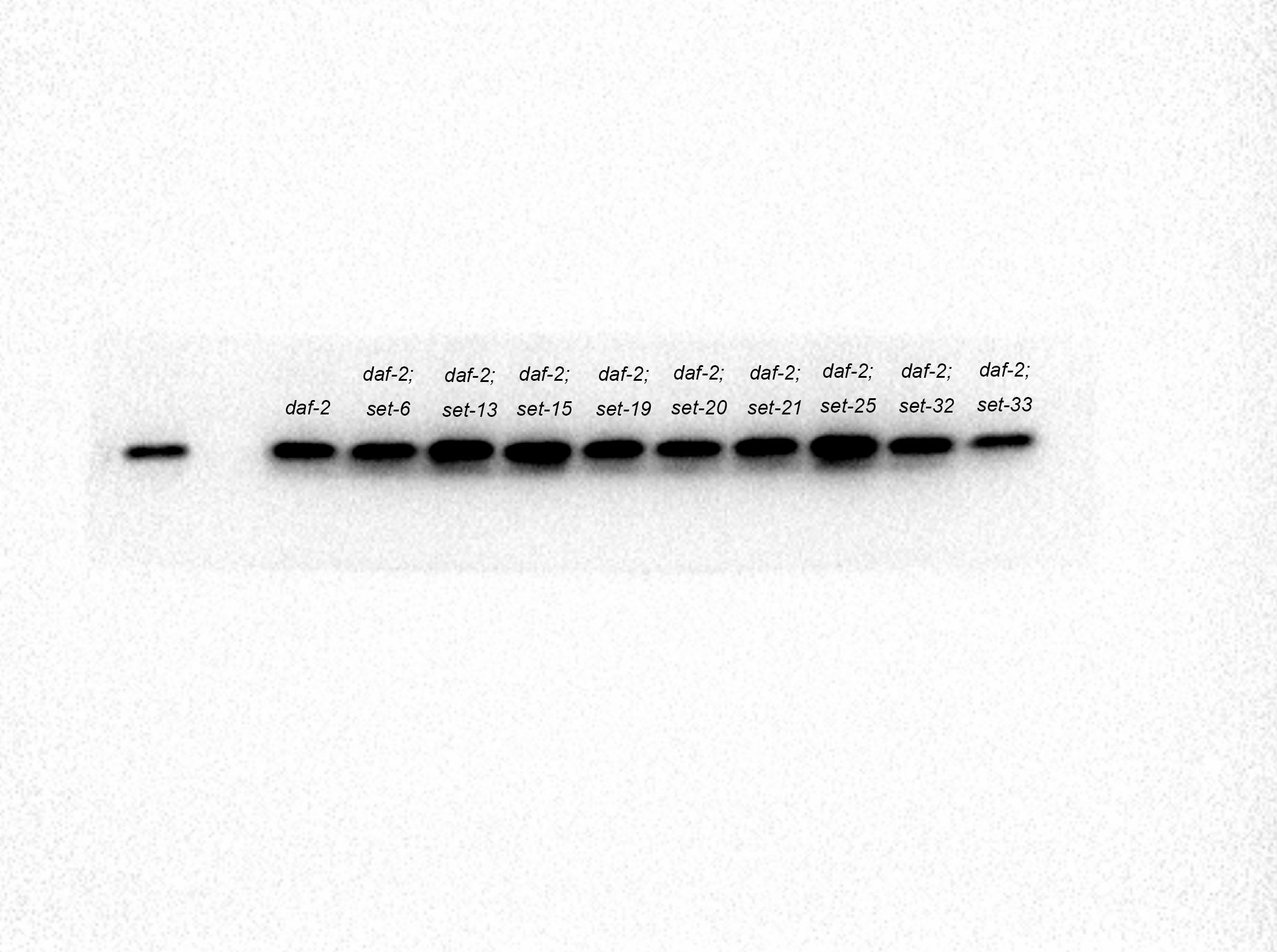

Supplement: Figure 8—source data 1. [file elife-74812-fig8-data1.zip › source data 2/figure8A/H3K4me3.tif]

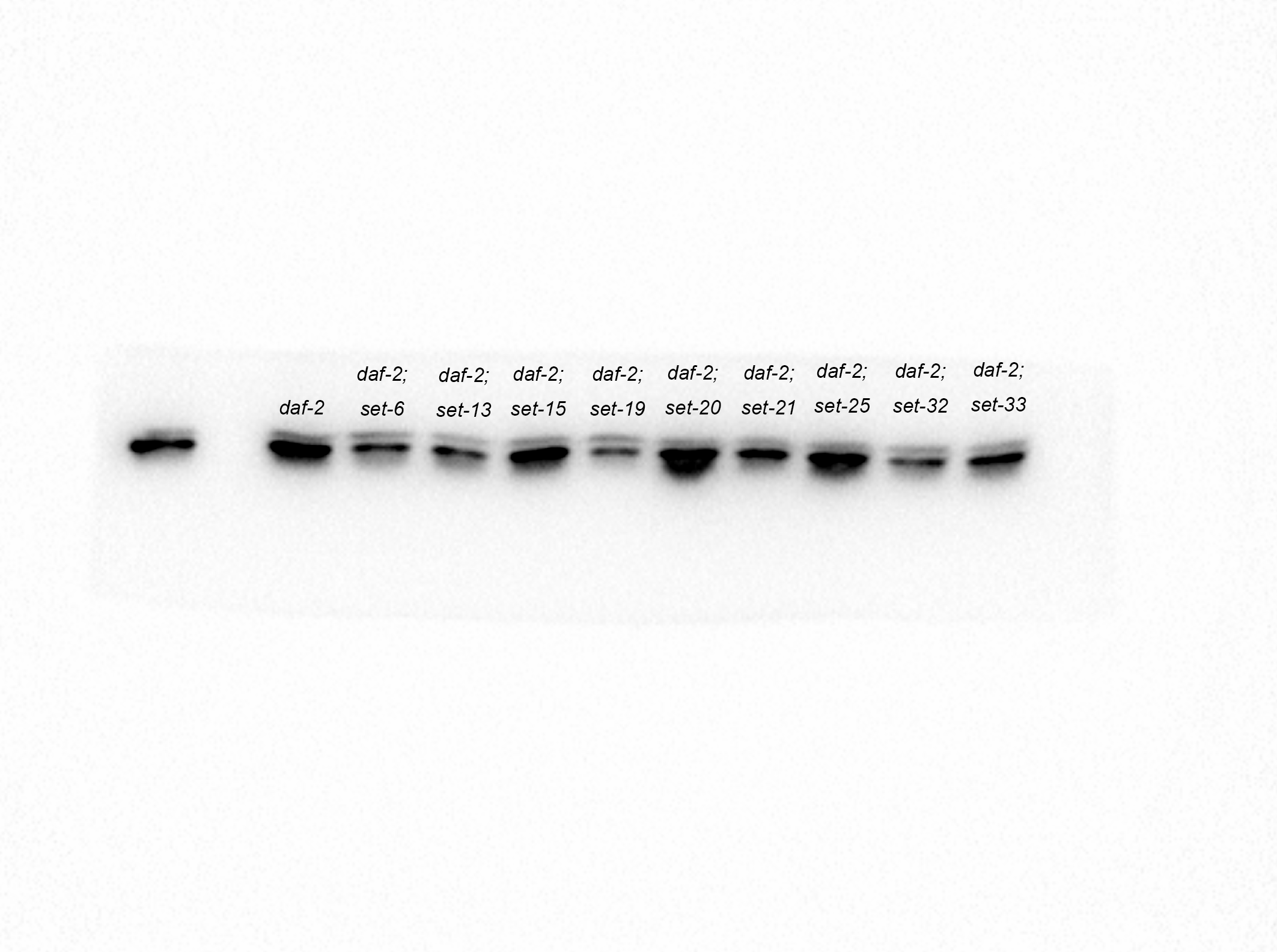

Supplement: Figure 8—source data 1. [file elife-74812-fig8-data1.zip › source data 2/figure8A/H3K9me1-1.tif]

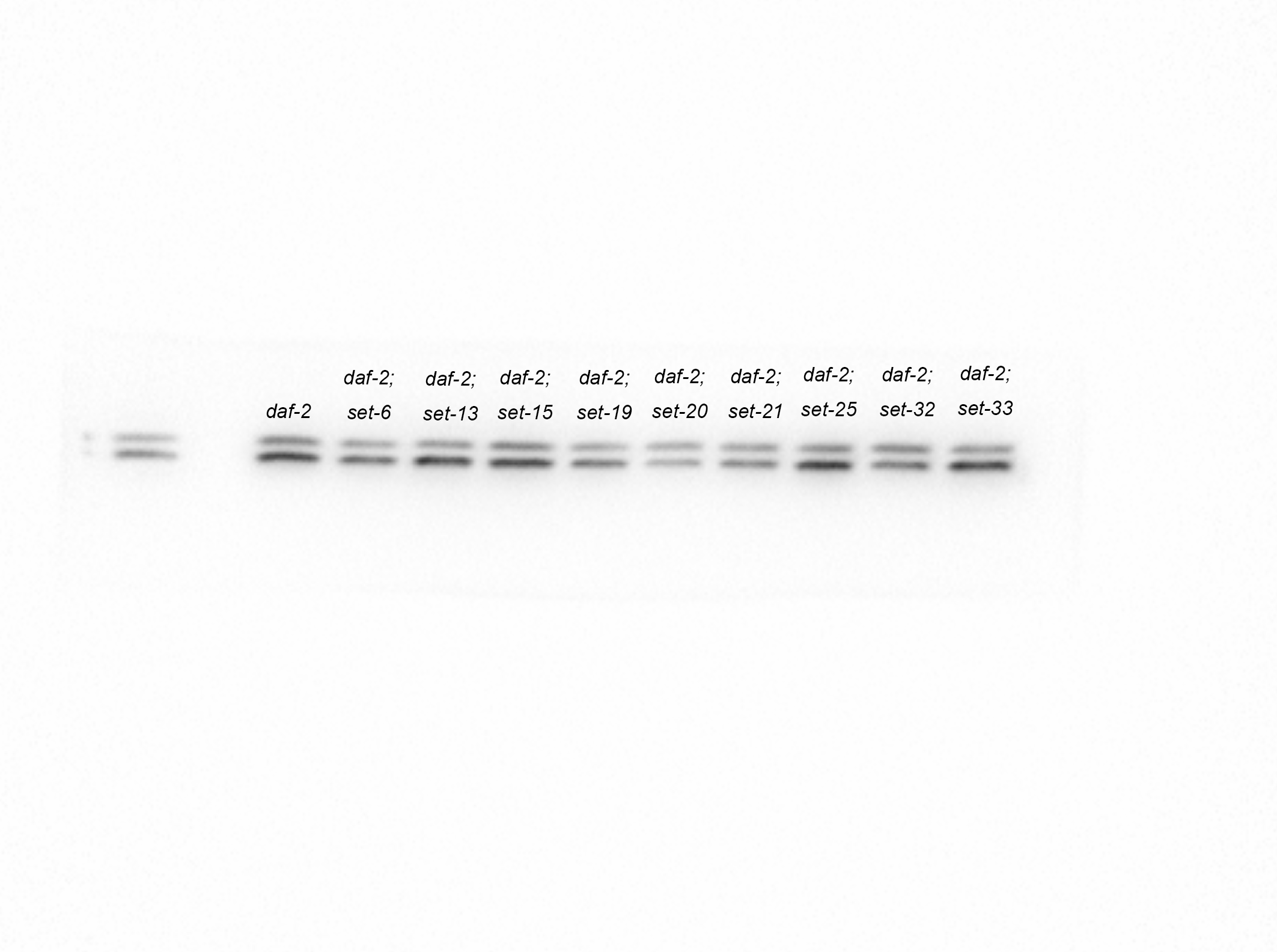

Supplement: Figure 8—source data 1. [file elife-74812-fig8-data1.zip › source data 2/figure8A/H3K9me1-2.tif]

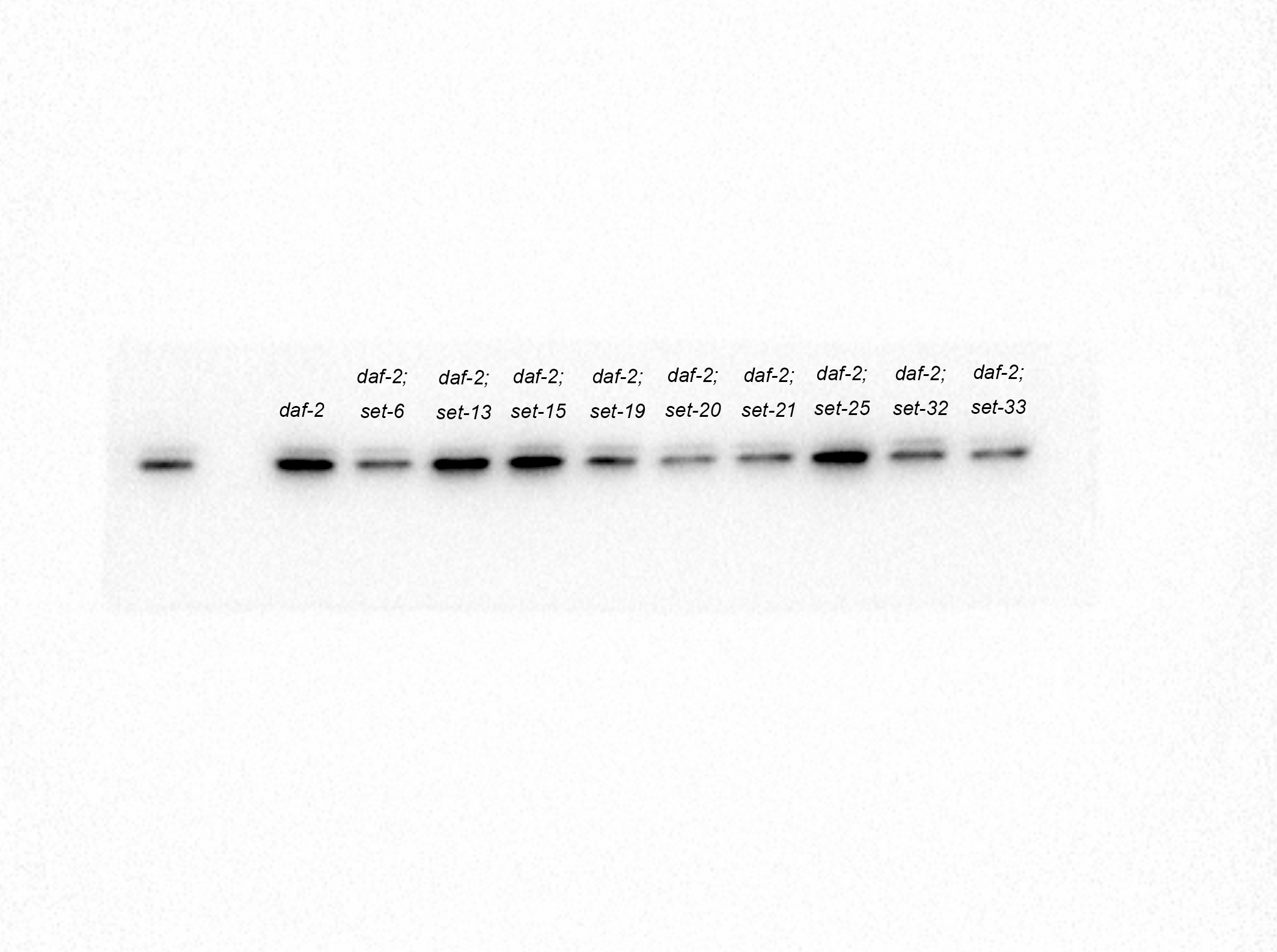

Supplement: Figure 8—source data 1. [file elife-74812-fig8-data1.zip › source data 2/figure8A/H3K9me1-3.tif]

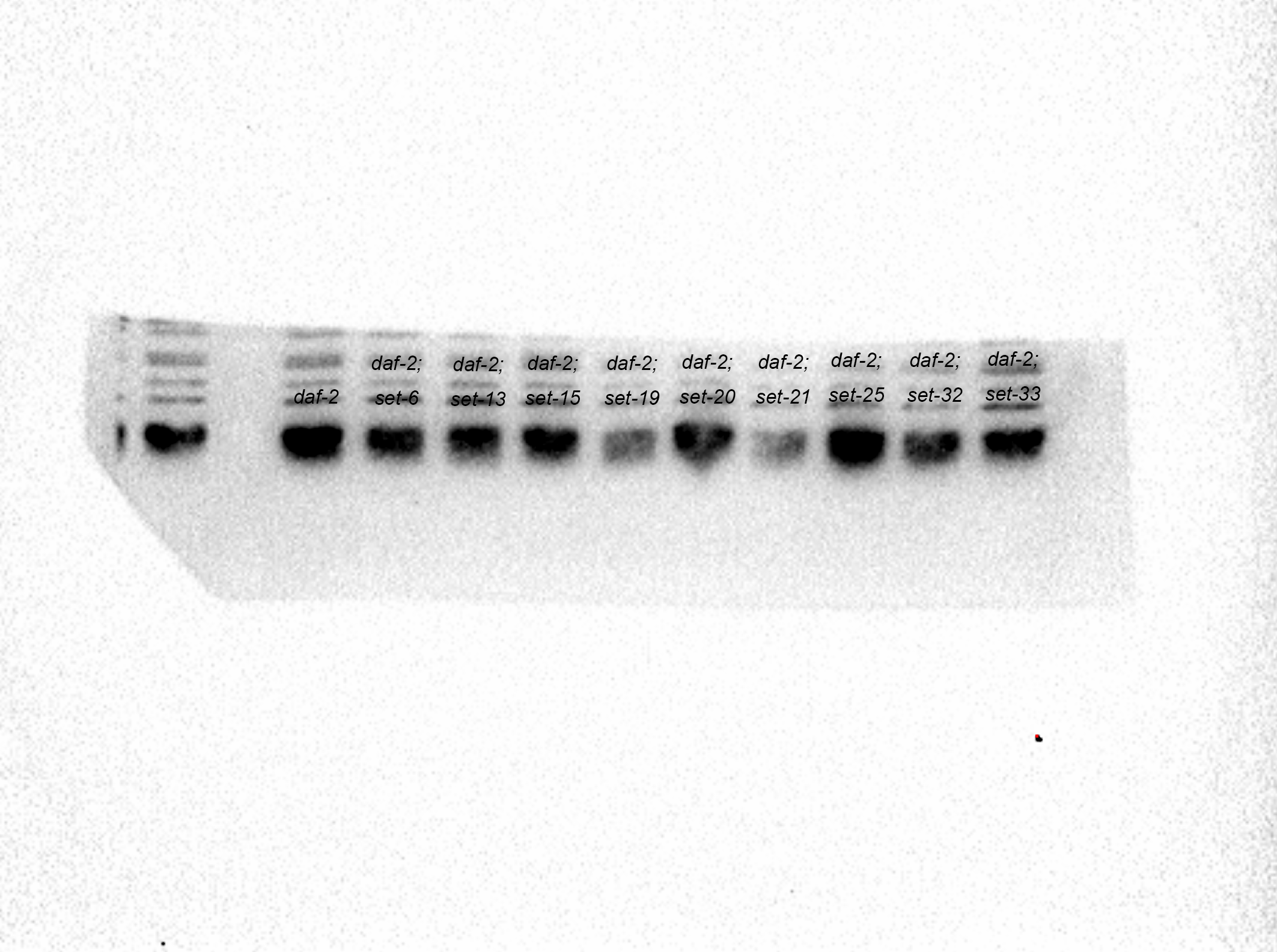

Supplement: Figure 8—source data 1. [file elife-74812-fig8-data1.zip › source data 2/figure8A/H3K9me2-1.tif]

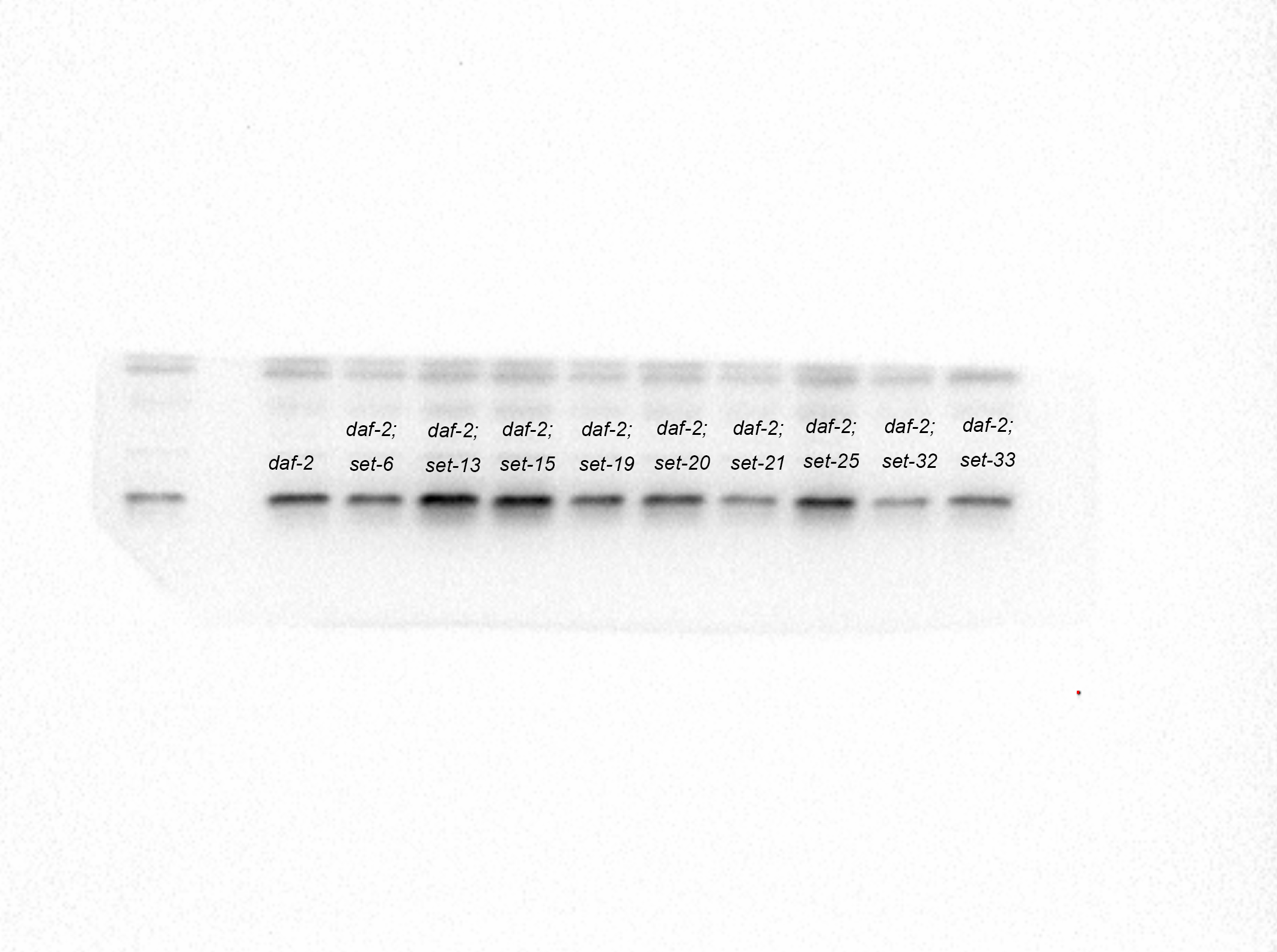

Supplement: Figure 8—source data 1. [file elife-74812-fig8-data1.zip › source data 2/figure8A/H3K9me2-2.tif]

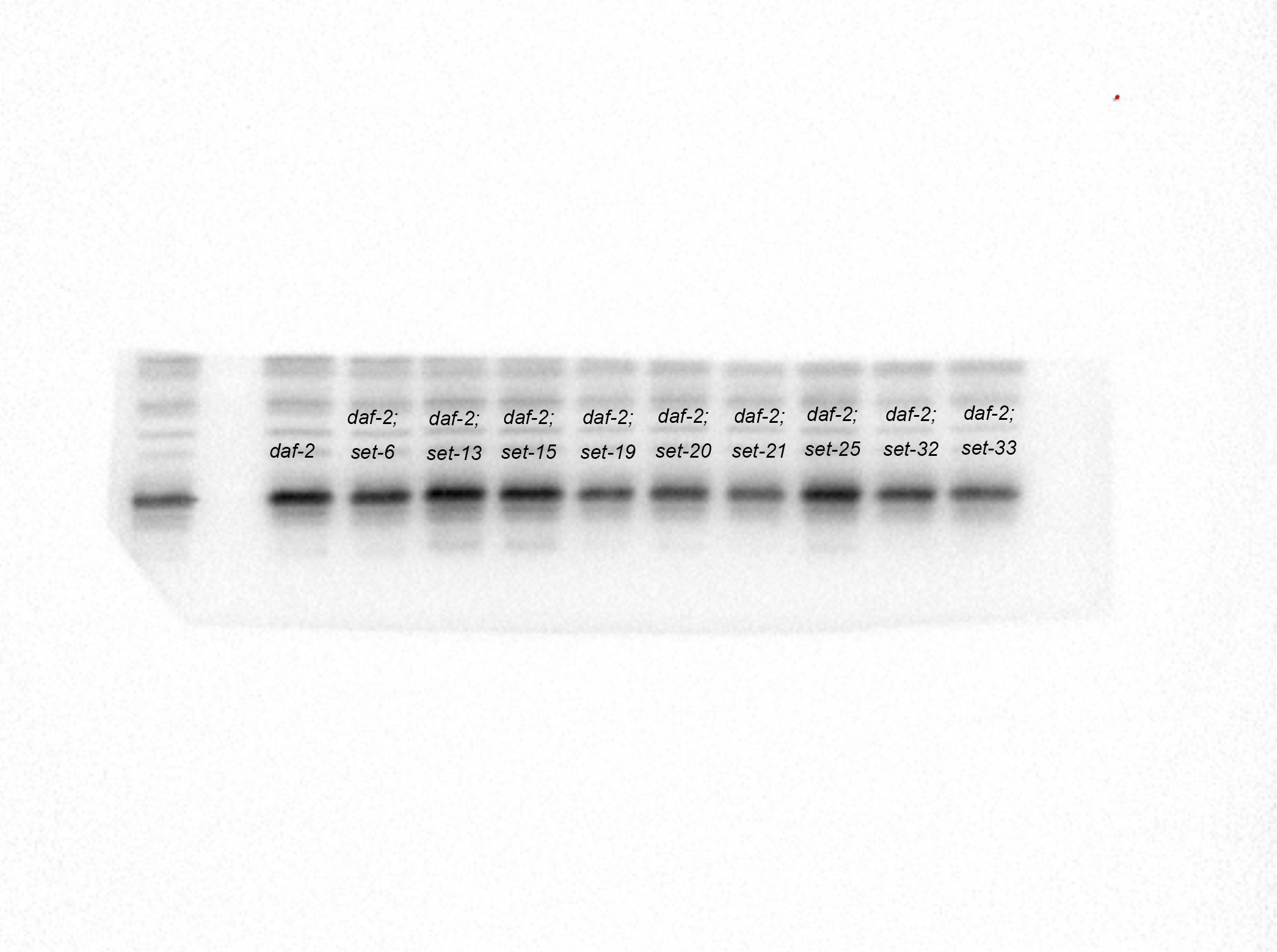

Supplement: Figure 8—source data 1. [file elife-74812-fig8-data1.zip › source data 2/figure8A/H3K9me2-3.tif]

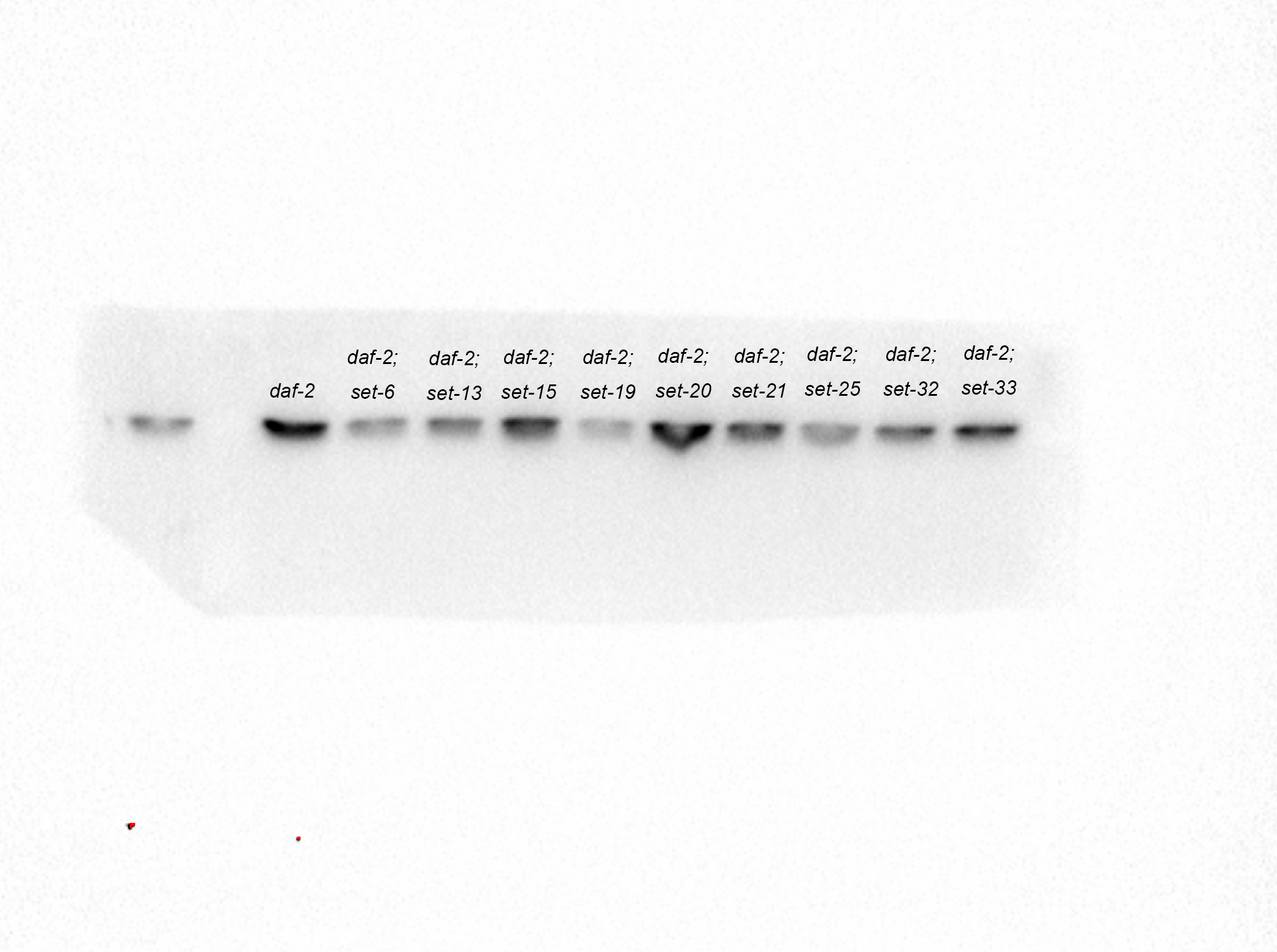

Supplement: Figure 8—source data 1. [file elife-74812-fig8-data1.zip › source data 2/figure8A/H3K9me3-1.tif]

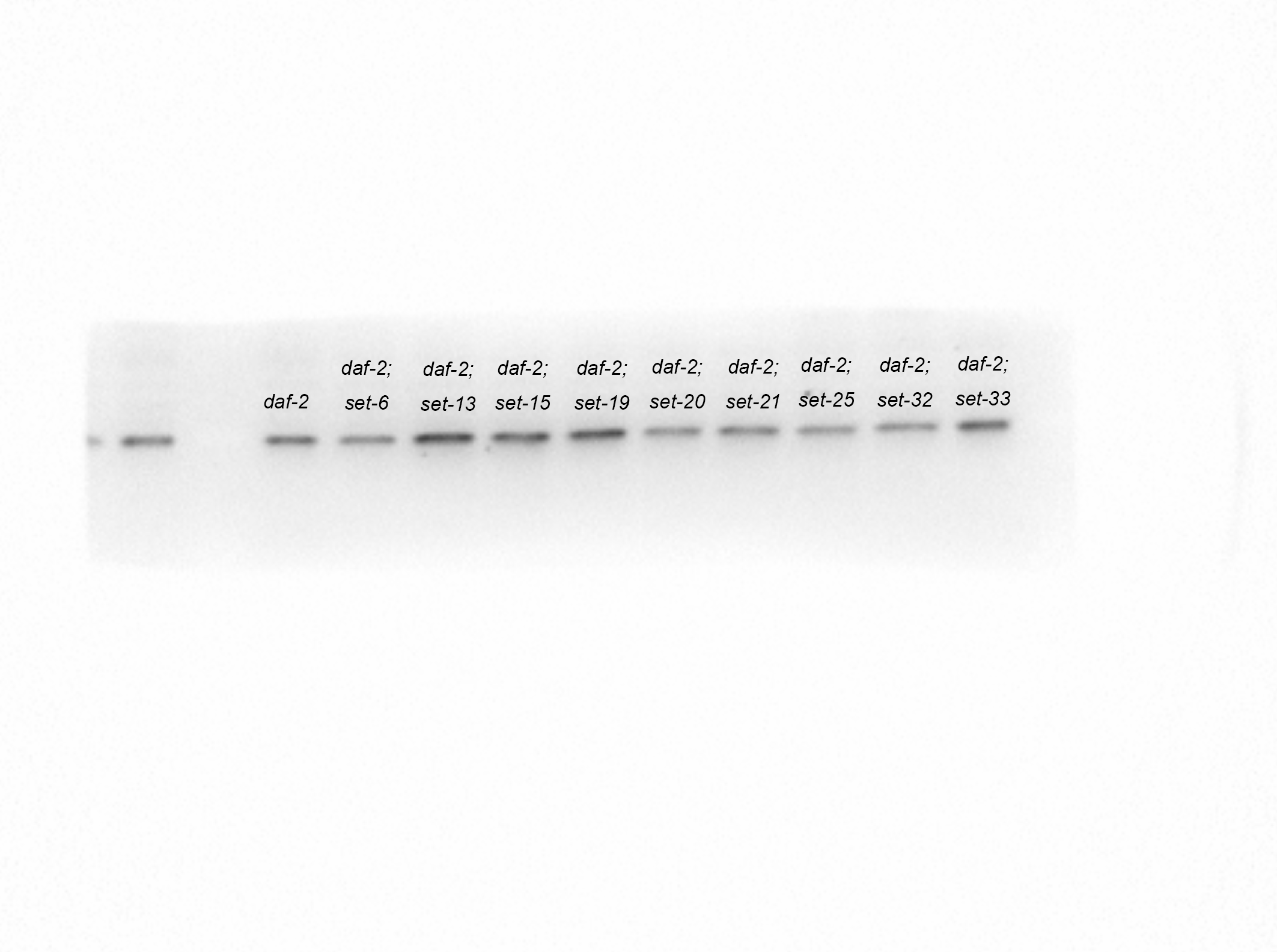

Supplement: Figure 8—source data 1. [file elife-74812-fig8-data1.zip › source data 2/figure8A/H3K9me3-2.tif]

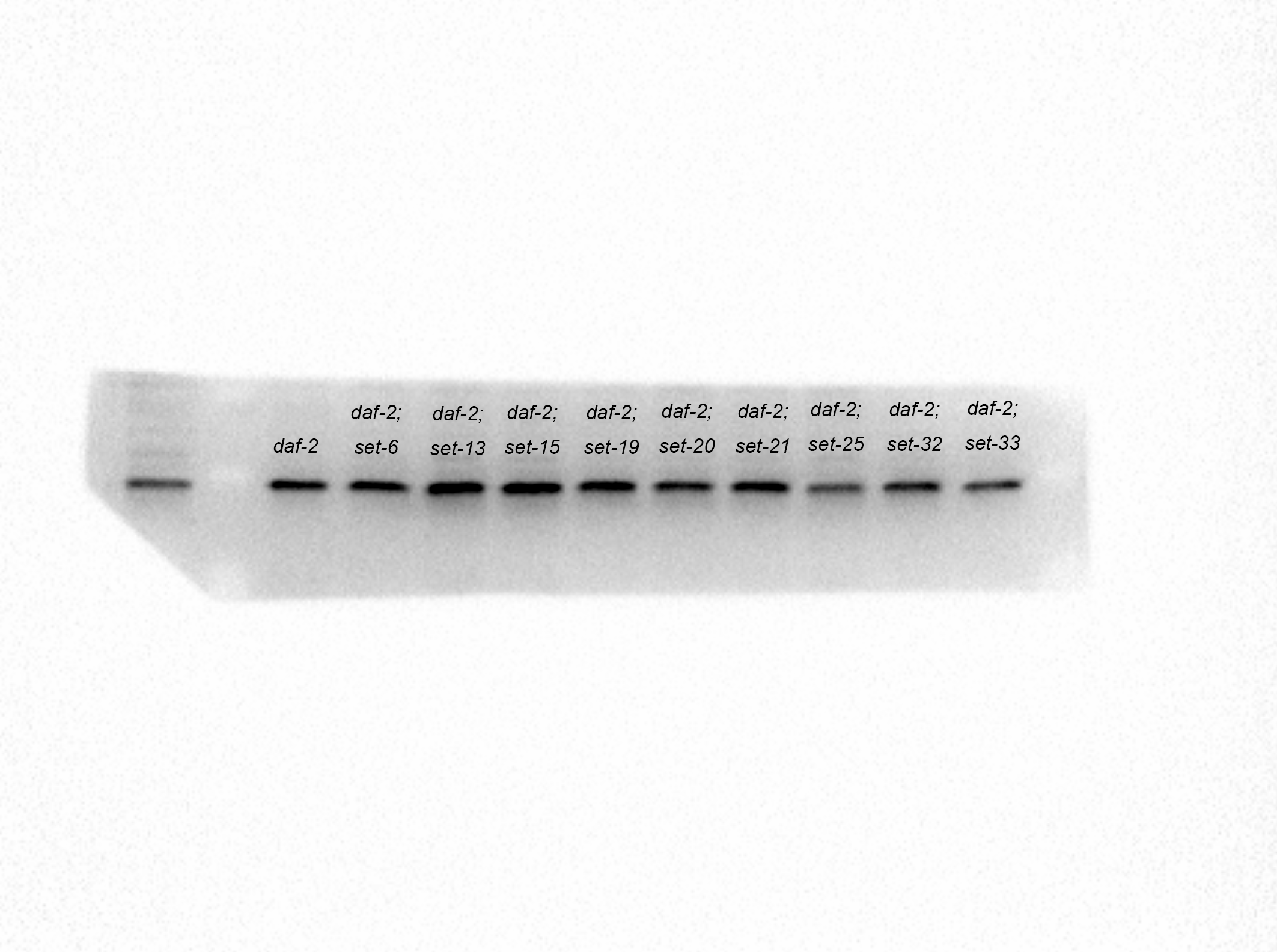

Supplement: Figure 8—source data 1. [file elife-74812-fig8-data1.zip › source data 2/figure8A/H3K9me3-3.tif]

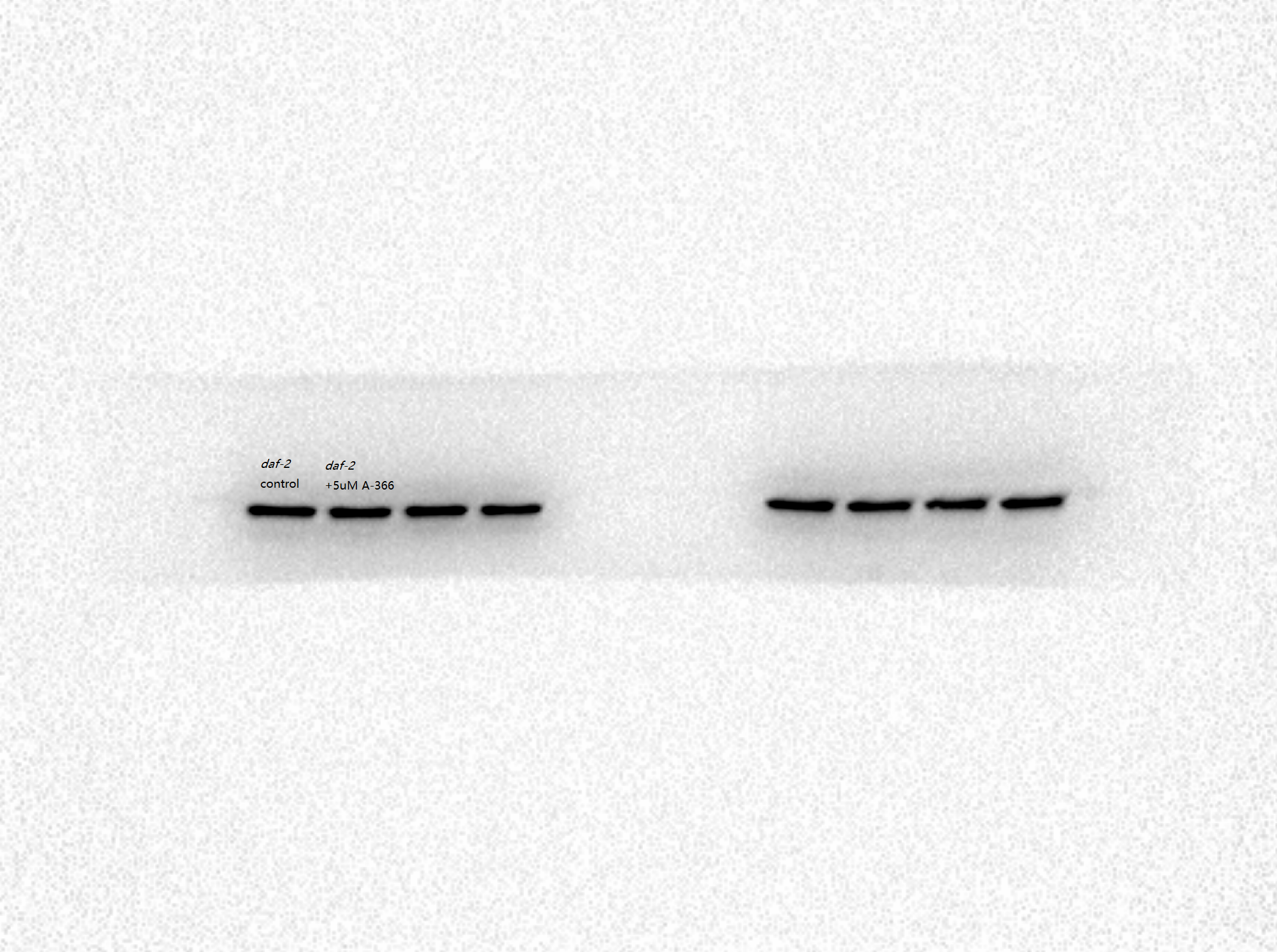

Supplement: Figure 8—source data 1. [file elife-74812-fig8-data1.zip › source data 2/figure8B/replicate 1/Actin.tif]

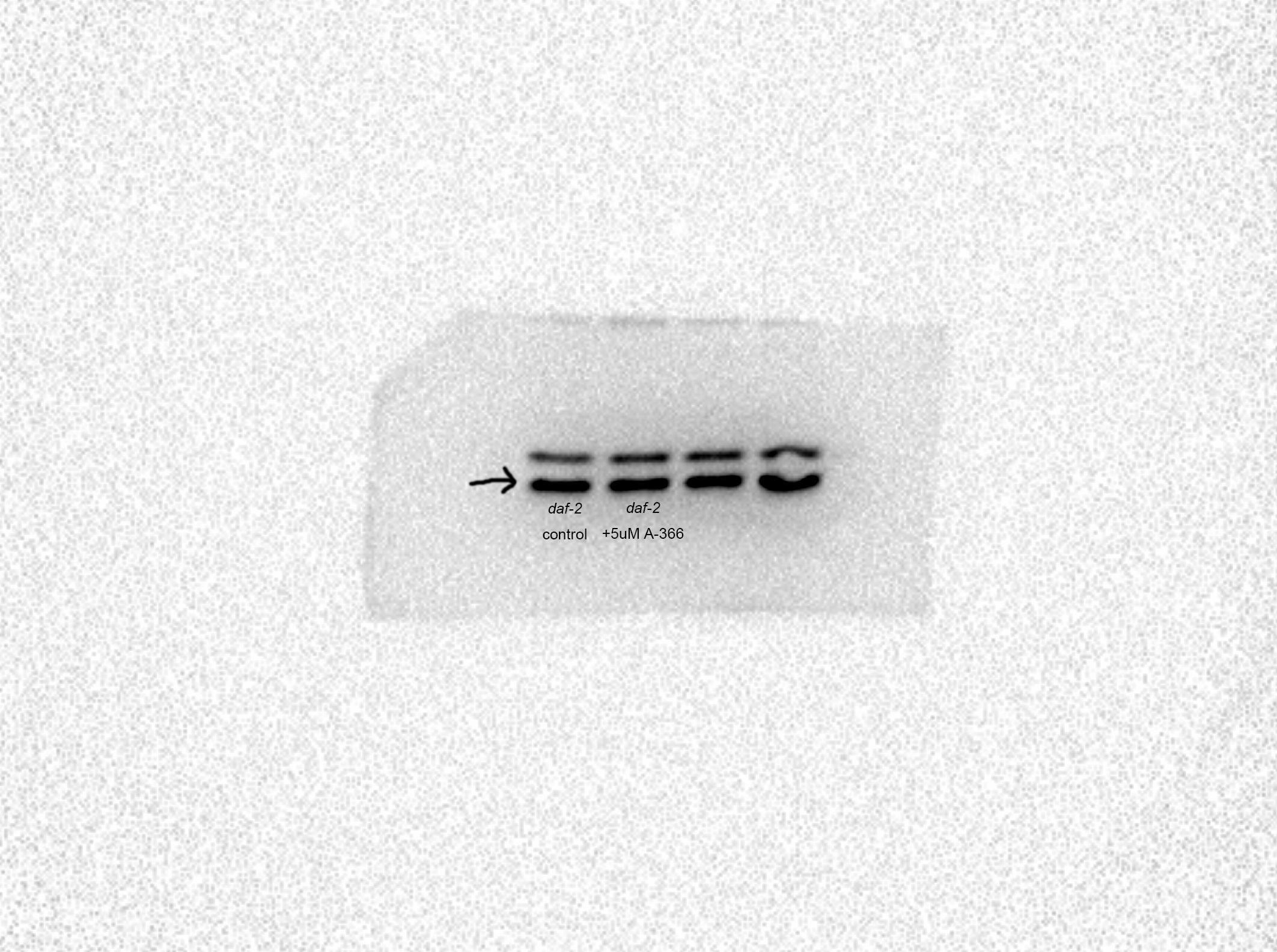

Supplement: Figure 8—source data 1. [file elife-74812-fig8-data1.zip › source data 2/figure8B/replicate 1/H3K9me1.tif]

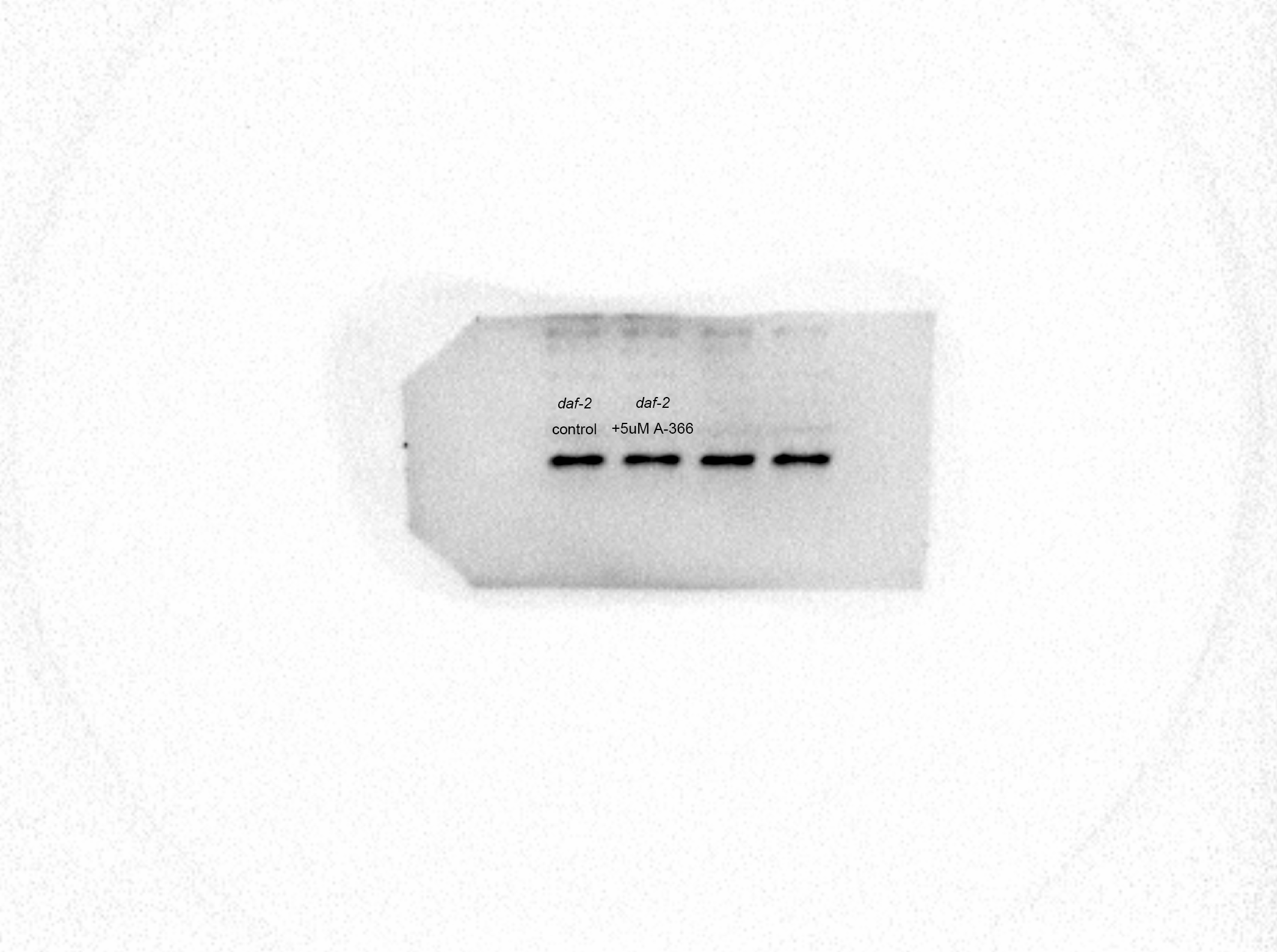

Supplement: Figure 8—source data 1. [file elife-74812-fig8-data1.zip › source data 2/figure8B/replicate 1/H3K9me2.tif]

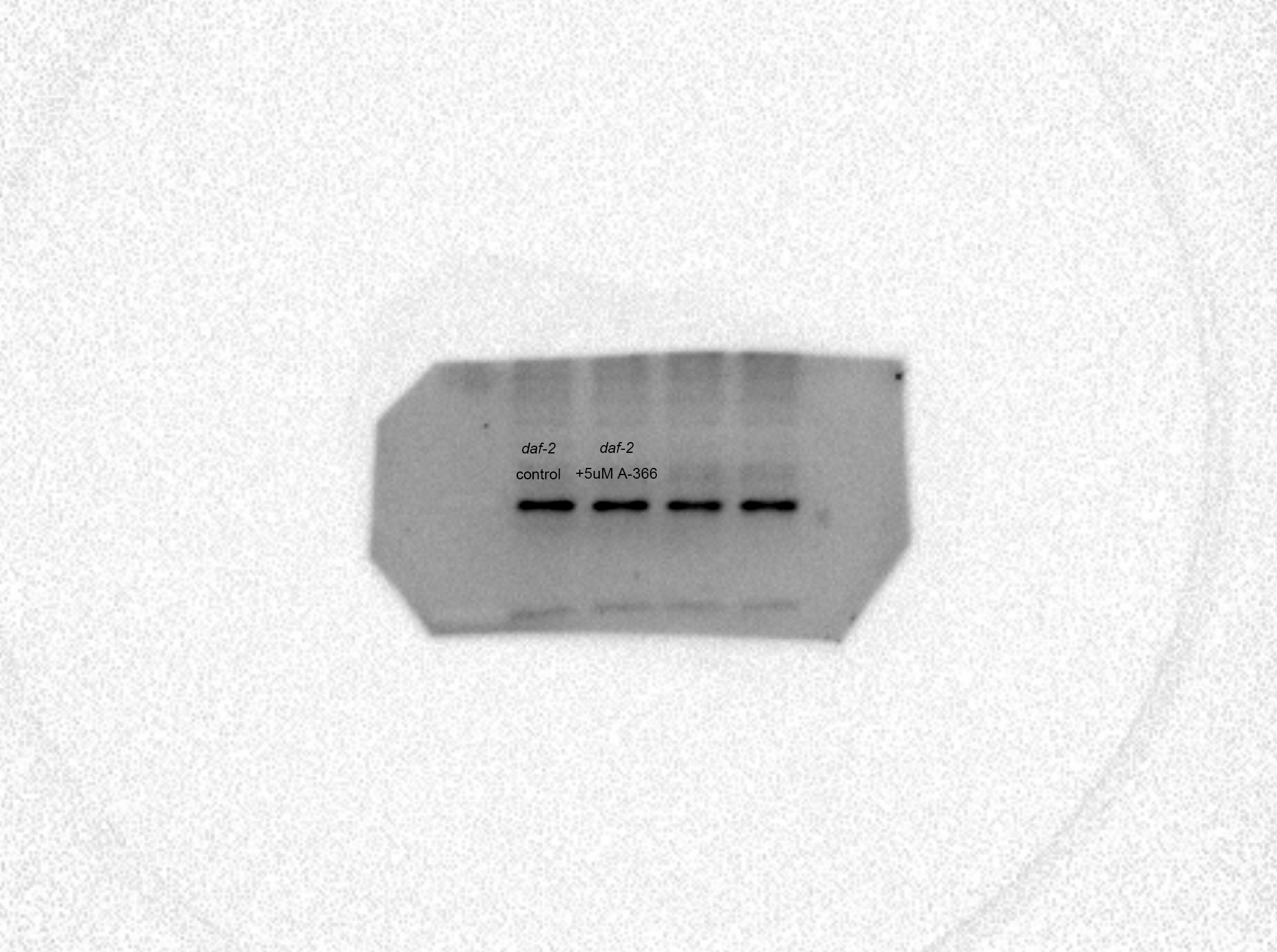

Supplement: Figure 8—source data 1. [file elife-74812-fig8-data1.zip › source data 2/figure8B/replicate 1/H3K9me3.tif]

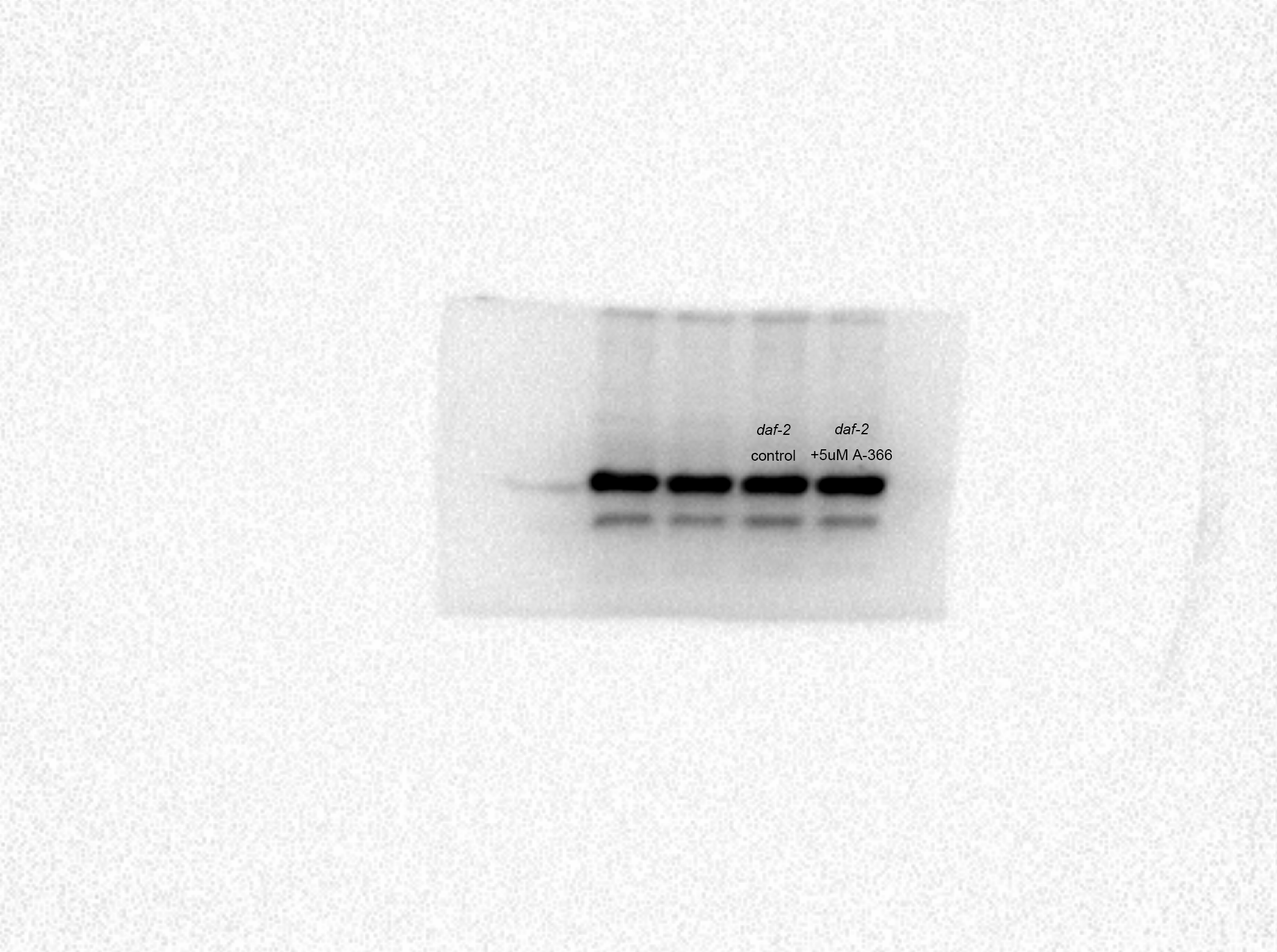

Supplement: Figure 8—source data 1. [file elife-74812-fig8-data1.zip › source data 2/figure8B/replicate 1/H3.tif]

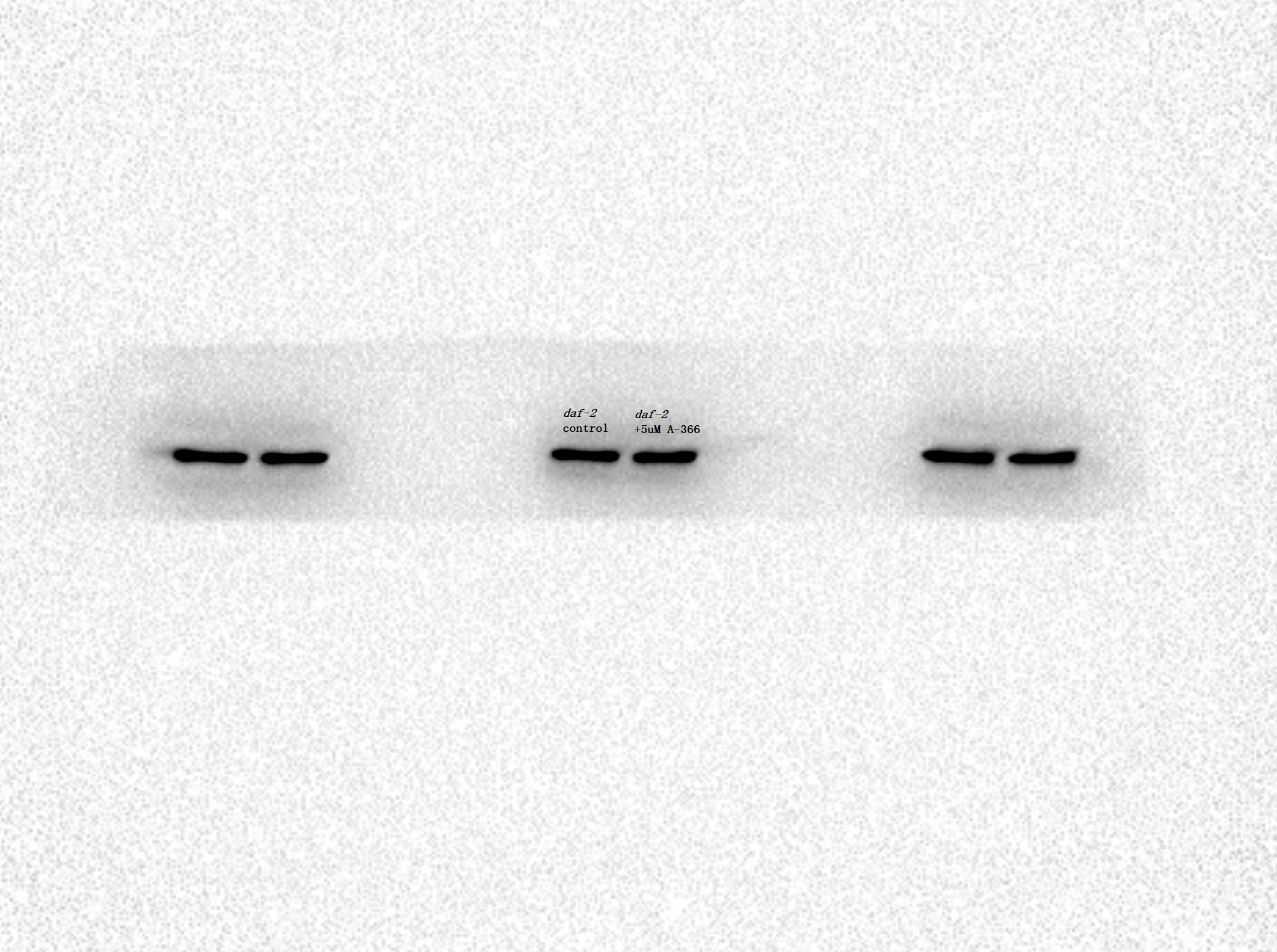

Supplement: Figure 8—source data 1. [file elife-74812-fig8-data1.zip › source data 2/figure8B/replicate 2/Actin.tif]

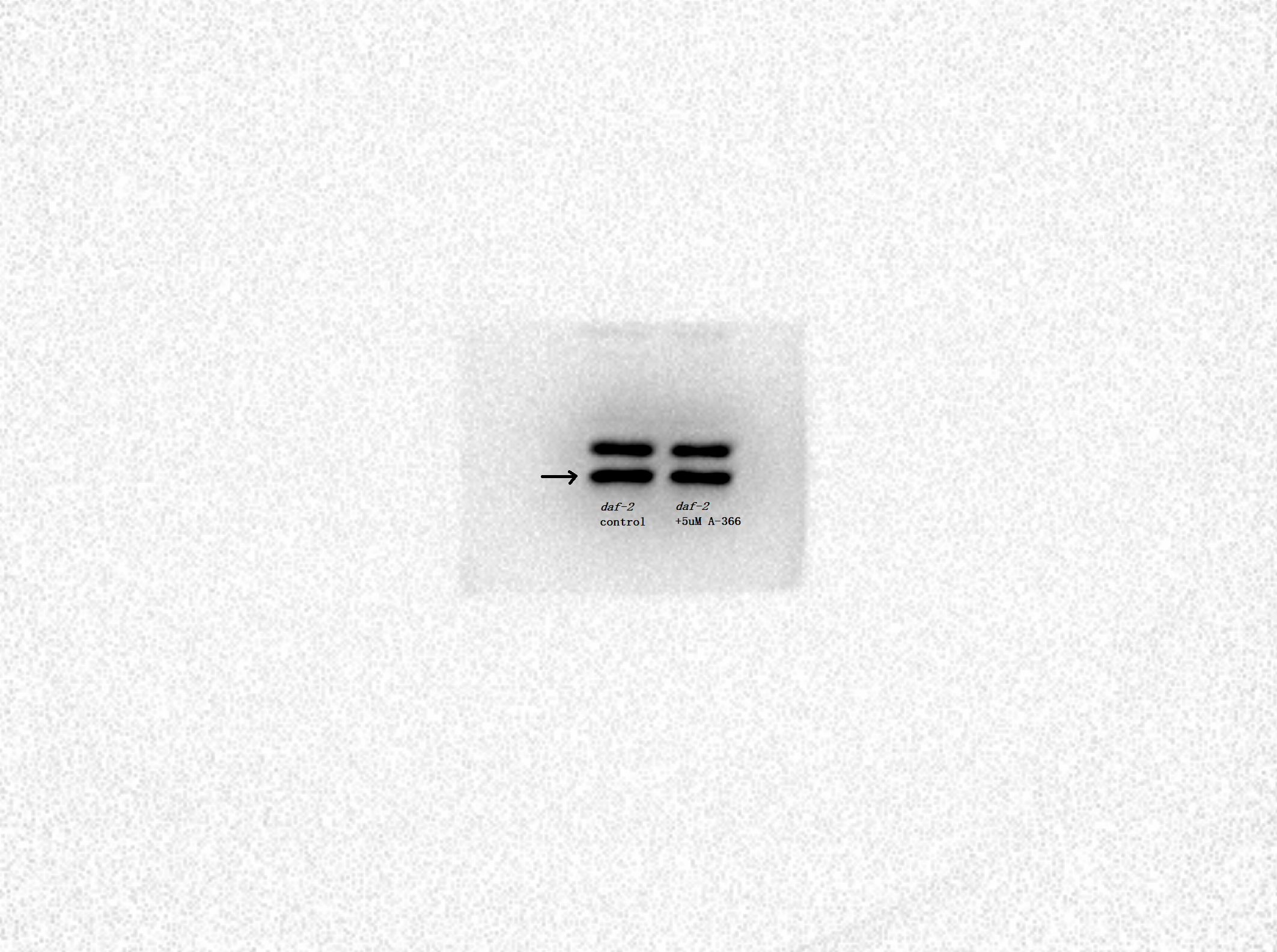

Supplement: Figure 8—source data 1. [file elife-74812-fig8-data1.zip › source data 2/figure8B/replicate 2/H3K9me1.tif]

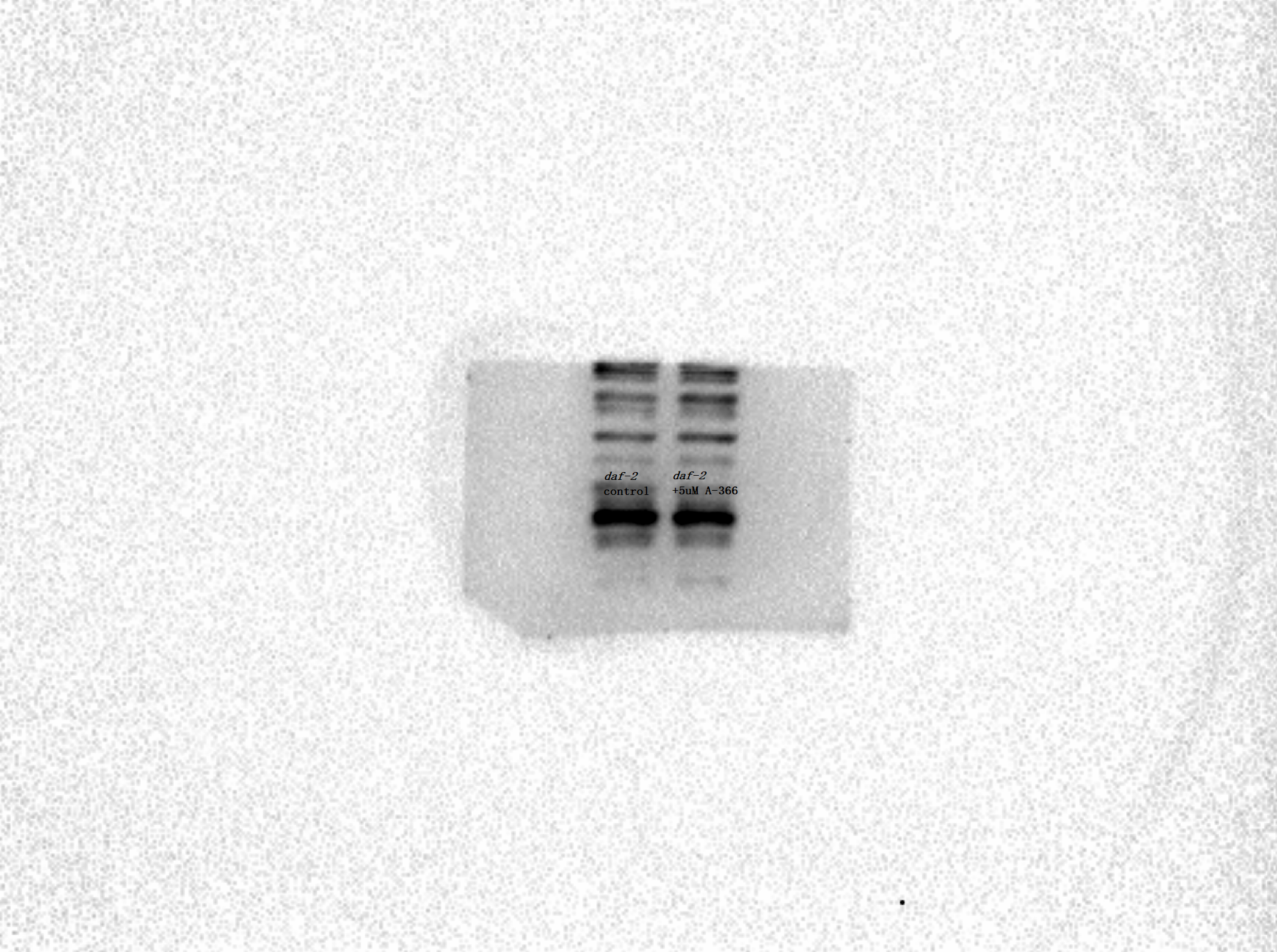

Supplement: Figure 8—source data 1. [file elife-74812-fig8-data1.zip › source data 2/figure8B/replicate 2/H3K9me2.tif]

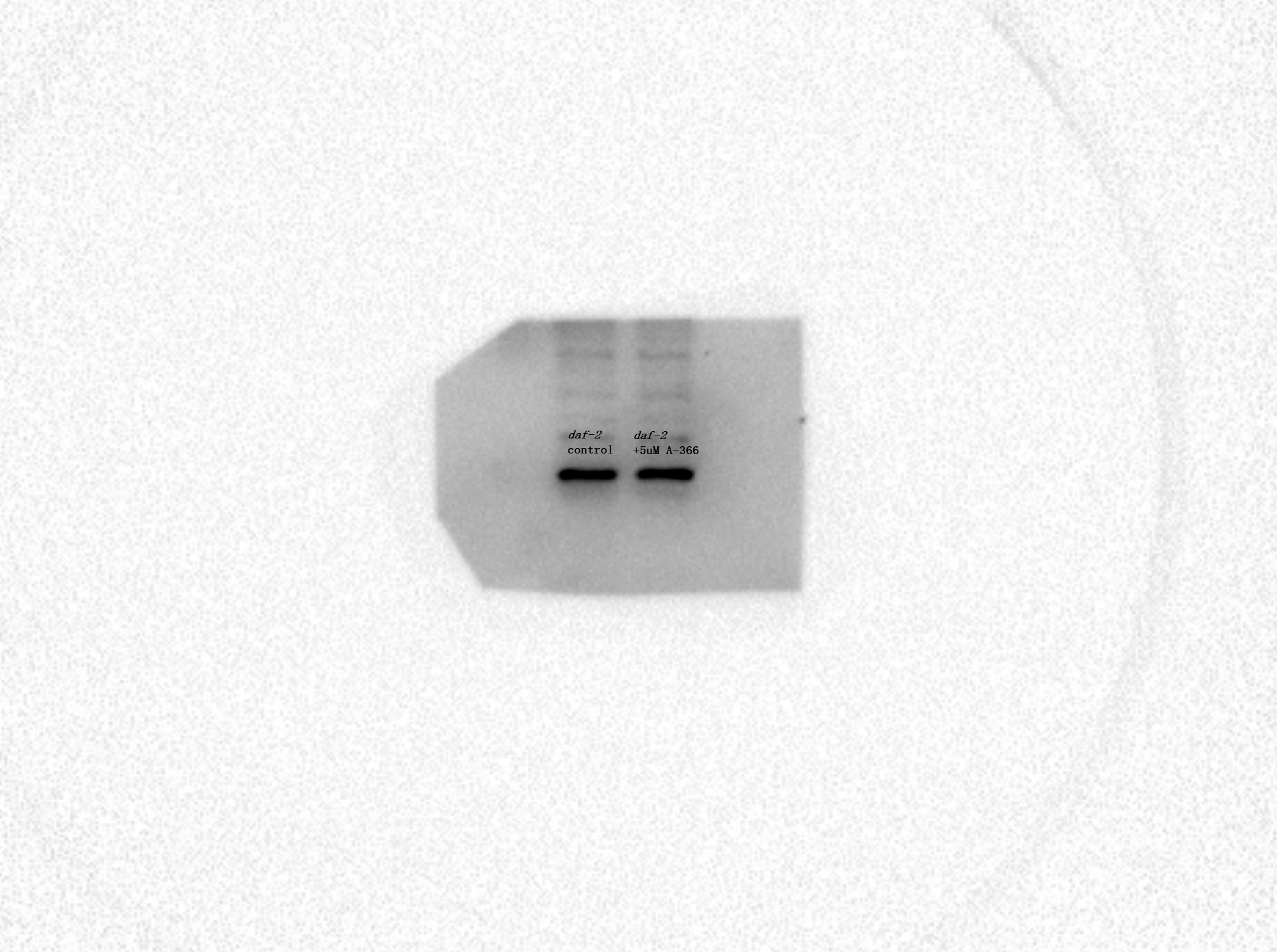

Supplement: Figure 8—source data 1. [file elife-74812-fig8-data1.zip › source data 2/figure8B/replicate 2/H3K9me3.tif]

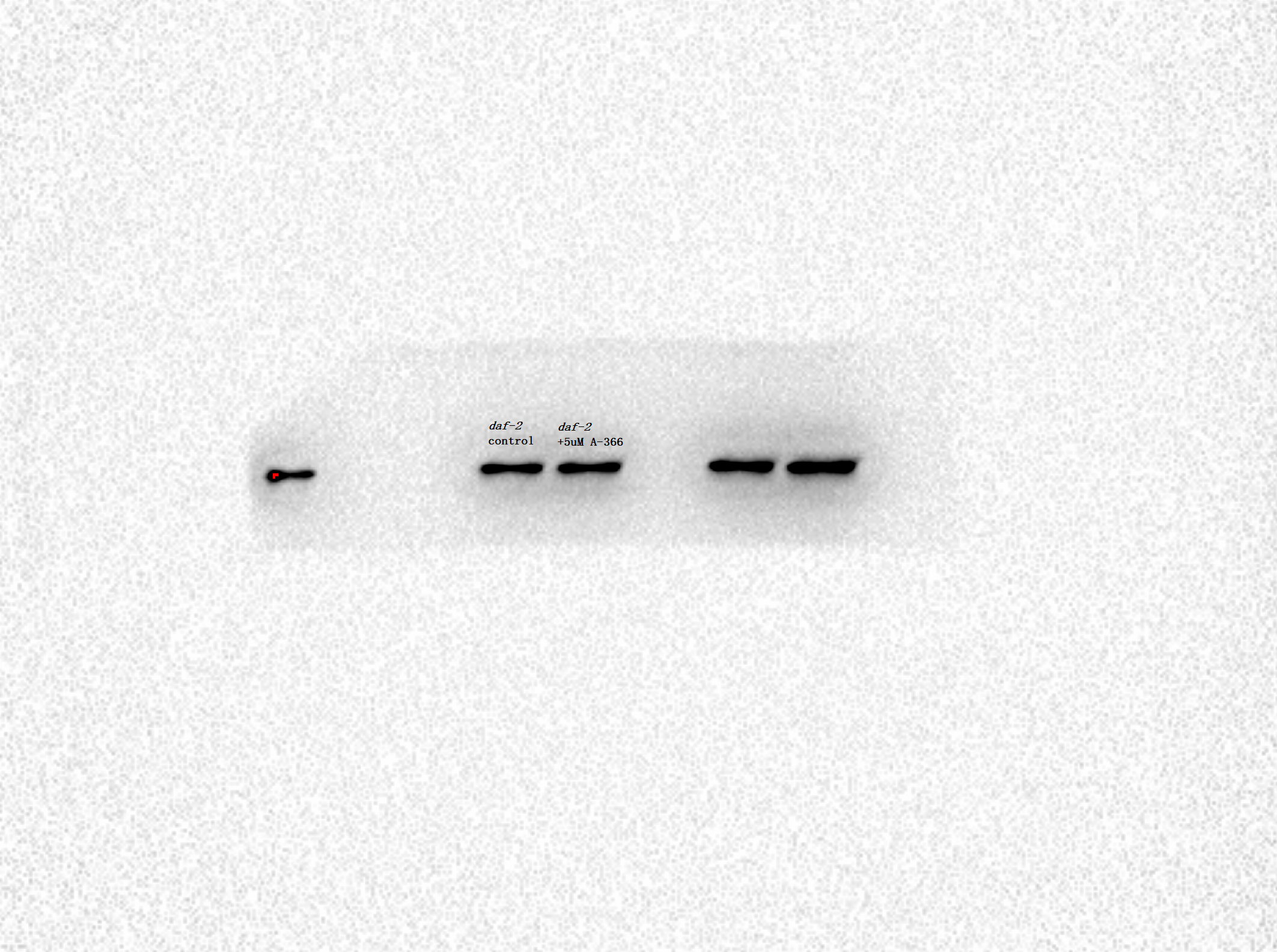

Supplement: Figure 8—source data 1. [file elife-74812-fig8-data1.zip › source data 2/figure8B/replicate 3/Actin.tif]

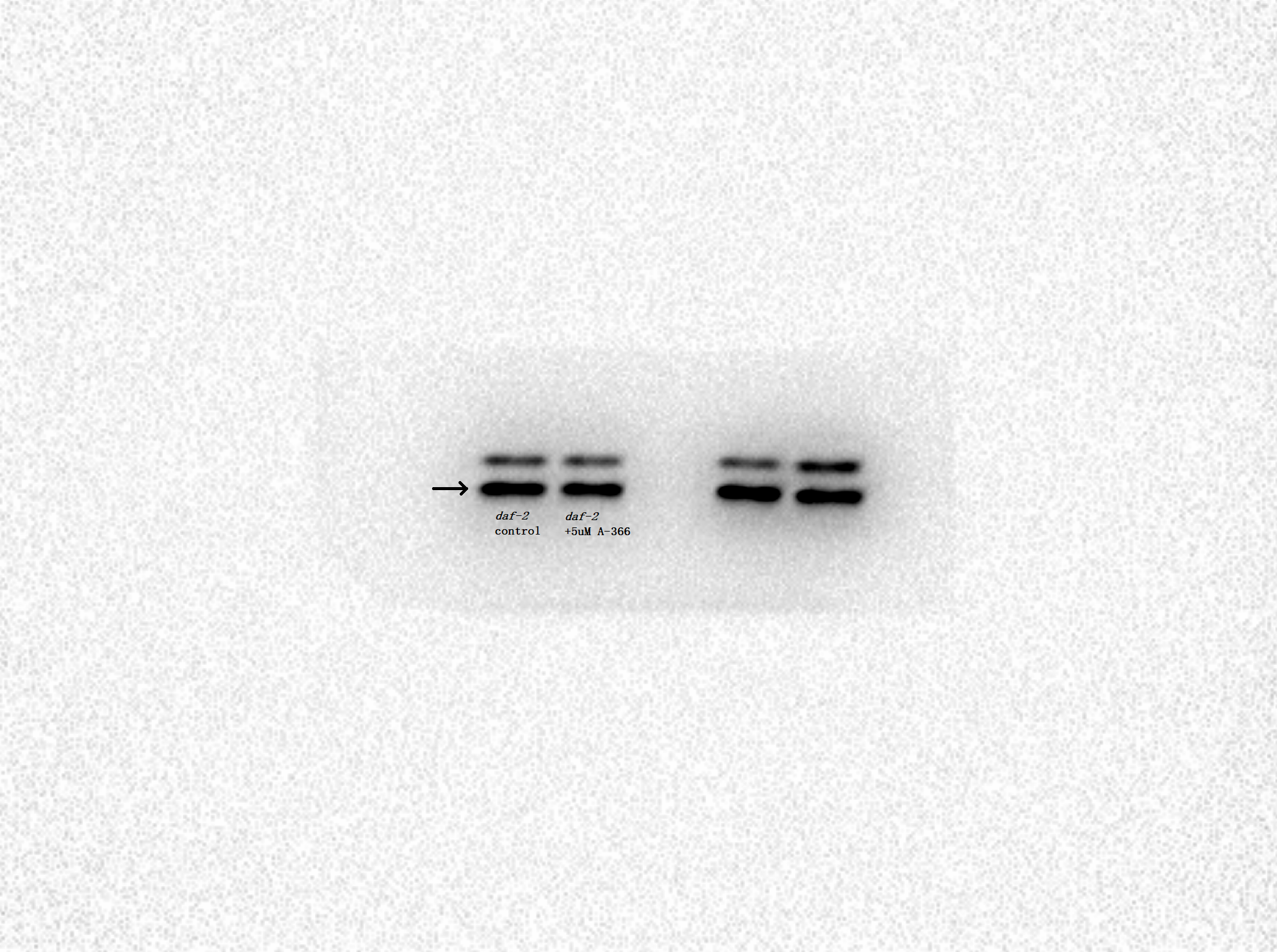

Supplement: Figure 8—source data 1. [file elife-74812-fig8-data1.zip › source data 2/figure8B/replicate 3/H3K9me1.tif]

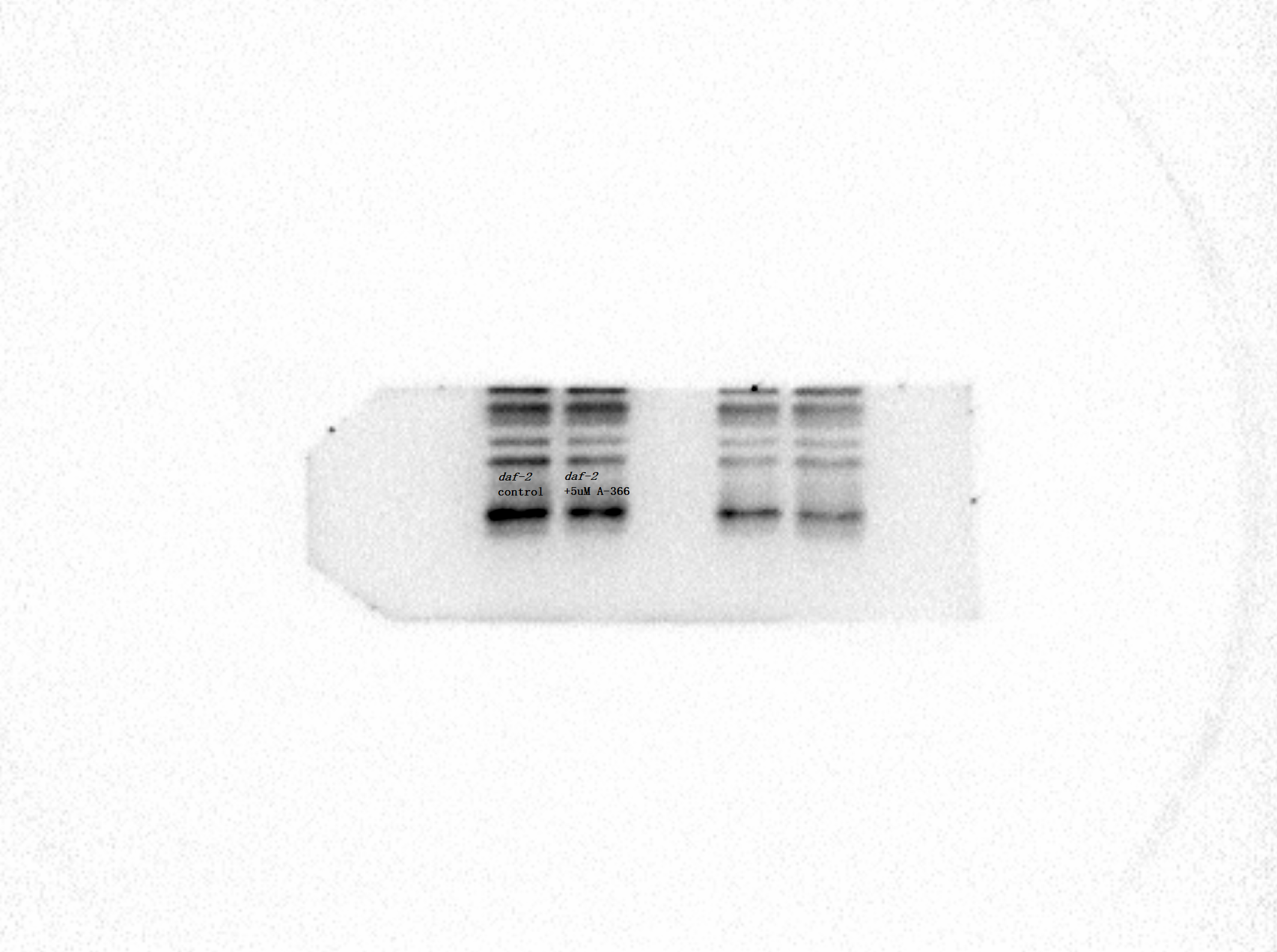

Supplement: Figure 8—source data 1. [file elife-74812-fig8-data1.zip › source data 2/figure8B/replicate 3/H3K9me2.tif]

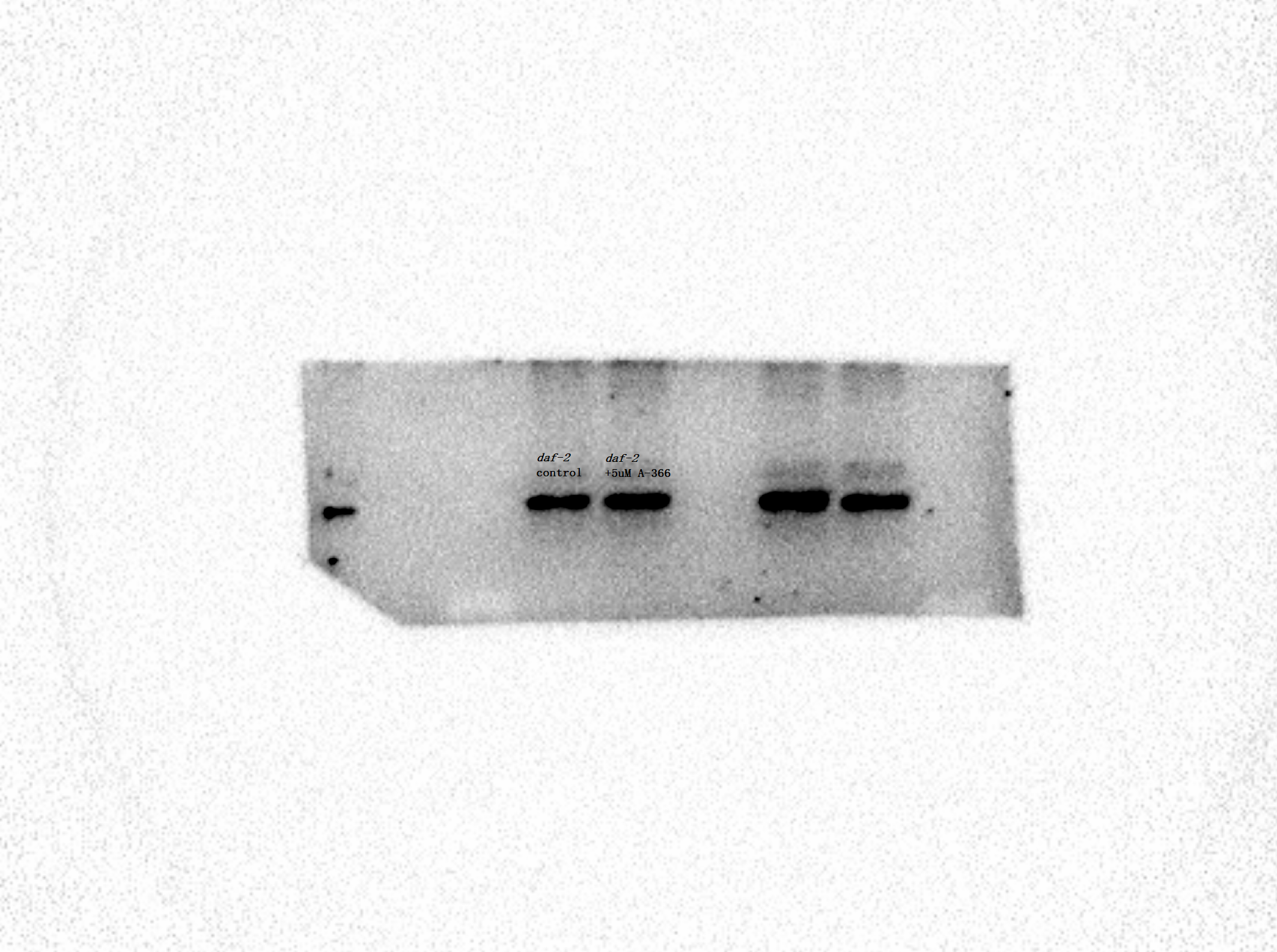

Supplement: Figure 8—source data 1. [file elife-74812-fig8-data1.zip › source data 2/figure8B/replicate 3/H3K9me3.tif]
