# Supplementary material for: H3K9me1/2 methylation limits the lifespan of daf-2 mutants in C. elegans
Source: eLife. 2022 Sep 20;11:e74812. doi: 10.7554/eLife.74812 (PMC9514849; doi:10.7554/eLife.74812)
Supplement: Supplementary file 2. [file elife-74812-supp2.docx]

**Supplementary file 2.** List of strains used in this study.

| **strain** | **genotype** |
| --- | --- |
| DA465 | *eat-2(ad465)* II |
| TJ356 | *zIs356 [daf-16p::daf-16a/b::GFP + rol-6(su1006)]* IV |
| RM1025 | *set-2(ok952)* III |
| VC2683 | *set-6(ok2195)* X |
| RB2406 | *set-15(ok3291)* IV |
| RB1517 | *set-19(ok1813)* X |
| RB1640 | *set-20(ok2022)* X |
| RB1793 | *set-21(ok2320)* IV |
| MT17463 | *set-25(n5021)* III |
| VC967 | *set-32(ok1457)* I |
| MT13293 | *met-2(n4256)* III |
| SHG463 | *set-13(ust54)* II |
| SHG563 | *daf-2(e1370)* III;*set-21(ok2320)* IV |
| SHG564 | *daf-2(e1370)* III;*set-13(ust54)* II |
| SHG565 | *daf-2(e1370)* III;*daf-16(mu86)* I |
| SHG728 | *set-6(ok2195)* X;*daf-2(e1370)* III |
| SHG729 | *set-19(ok1813)* X;*daf-2(e1370)* III |
| SHG730 | *set-20(ok2022*) X;*daf-2(e1370)* III |
| SHG731 | *set-25(n5021)* III;*daf-2(e1370)* III |
| SHG732 | *set-32(ok1457)* I;*daf-2(e1370)* III |
| SHG733 | *rsks-1(ok1255)* III;*set-21(ust68)* IV |
| SHG734 | *rsks-1(ok1255)* III;*daf-2(e1370)* III;*set-21(ust68)* IV |
| SHG792 | *daf-2(e1370)* III;*daf-16(mu86)* I;*set-21(ust68)* IV |
| SHG822 | *set-21(ust68)* IV;*zIs356 [daf-16p::daf-16a/b::GFP + rol-6(su1006)]* IV |
| SHG823 | *set-25(n5021)* III;*zIs356 [daf-16p::daf-16a/b::GFP + rol-6(su1006)]* IV |
| SHG841 | *daf-2(e1370)* III;*hpl-2(tm1489)* III |
| SHG846 | *daf-2(e1370)* III;*set-21(ust68)* IV;*set-6(ok2195)* X |
| SHG847 | *daf-2(e1370)* III;*set-21(ust68)* IV;*set-19(ok1813)* X |
| SHG848 | *daf-2(e1370)* III;*set-21(ust68)* IV;*set-20(ok2022)* X |
| SHG849 | *daf-2(e1370)* III;*set-21(ust68)* IV;*set-32(ok1457)* I |
| SHG858 | *set-21(ust104)* IV |
| SHG861 | *set-21(ust107)* IV |
| SHG869 | *set-33(ust114)* X |
| SHG983 | *ustIS204 3 x flag::gfp::set-21)* IV |
| SHG984 | *ustIS206(set-25::gfp:: 3 x flag)* III |
| SHG985 | *ustIS205(3 x flag::gfp::set-32)* I |
| SHG1128 | *ustIS62(set-13p::set-13::gfp:: 3 x flag)* IV |
| SHG1131 | *rsks-1(ok1255)* III;*daf-2(e1370)* III |
| SHG1133 | *daf-2(e1370)* III (3x) |
| SHG1136 | *daf-16(mu86)* I;*set-21(ust69)* IV |
| SHG1137 | *daf-2(e1370)* III;*set-21(ust69*) IV |
| SHG1138 | *set-21(ust68)* IV |
| SHG1139 | *set-21(ust69)* IV |
| SHG1140 | *daf-2(e1370)* III;*set-21(ust68)* IV |
| SHG1141 | *daf-16(mu86)* I;*set-21(ust68)* IV |
| SHG1145 | *daf-2(e1370)* III;*set-15(ok3291)* IV |
| SHG1146 | *daf-2(e1370*) III;*set-21(ust68)* IV;*set-2(ok952)* III |
| SHG1147 | *daf-2(e1370)* III;*set-2(ok952)* III |
| SHG1148 | *daf-2(e1370)* III;*hpl-1(tm1624)* X |
| SHG1149 | *daf-16(mu86)* I;*daf-2(e1370)* III;*set-21(ust68)* IV |
| SHG1150 | *daf-2(e1370)* III;*set-21(ust104)* IV |
| SHG1151 | *daf-2(e1370)* III;*set-33(ust114)* X |
| SHG1152 | *daf-2(e1370)* III;*3 x flag::gfp::set-32(ustIS205)* I |
| SHG1153 | *daf-2(e1370)* III;*3 x flag::gfp::set-21(ustIS204)* IV |
| SHG1154 | *daf-2(e1370)* III;*set-25::gfp:: 3 x flag(ustIS206)* III |
| SHG1164 | *daf-2(e1370)* III;*set-21(ust68) IV;asm-2(tm3746)* X |
| SHG1165 | *daf-2(e1370)* III;*set-21(ust68)* IV;*ins-35(ok3297)* V |
| SHG1166 | *daf-2(e1370)* III;*set-21(ust68)* IV;*lys-7(ok1384)* V |
| SHG1167 | *daf-2(e1370)* III;*set-21(ust68)* IV;*nhr-62(tm1818)* I |
| SHG1168 | *daf-2(e1370)* III;*set-21(ust68)* IV;*spp-12(tm2963)* V |
| SHG1169 | *daf-2(e1370)* III;*set-21(ust68)* IV;*tts-1(gk105)* X |
| SHG1170 | *daf-2(e1370)* III;*set-21(ust68)* IV;*sod-3(tm760)* X |
| SHG1171 | *daf-2(e1370)* III;*set-21(ust68)* IV;*dao-3(ok1678)* X |
| SHG1172 | *daf-2(e1370)* III;*set-21(ust68)* IV;*F35E8.7(ust198)* V |
| SHG1173 | *daf-2(e1370)* III;*set-21(ust68)* IV;*Y39G8B.7(ust197)* II |
| SHG1175 | *daf-16(mu86)* I;*set-2(ok952)* III |
| SHG1177 | *daf-2(e1370)* III;*set-21(ust68)* IV;*set-33(ust114)* X |
| SHG1178 | *daf-2(e1370)* III;*set-21(ust106)* IV |
| SHG1179 | *daf-2(e1370)* III;*set-21(ust107)* IV |
| SHG1199 | *met-2(n4256)* III;*daf-2(e1370)* III |
| SHG1367 | *daf-2(e1370)* III;*zIs356 [daf-16p::daf-16a/b::GFP + rol-6(su1006)]* IV |
| SHG1368 | *daf-2(e1370)* III;*set-25(n5021)* III  *zIs356 [daf-16p::daf-16a/b::GFP + rol-6(su1006)]* IV |
| SHG1369 | *daf-2(e1370)* III;*set-21(ust68)* IV  *zIs356 [daf-16p::daf-16a/b::GFP + rol-6(su1006)] I*V |
| SHG1785 | *eat-2(ad465)* II;*set-21(ust68)* IV |
| SHG1891 | *ustIS234(daf-16::gfp::3 x flag)* I |
| SHG1892 | *set-21(ust68)* IV;*ustIS234(daf-16::gfp::3 x flag)* I |
| SHG1893 | *set-25(n5021)* III;*ustIS234(daf-16::gfp::3 x flag)* I |
| SHG1894 | *daf-2(e1370)* III;*ustIS234(daf-16::gfp::3 x flag)* I |
| SHG1895 | *daf-2(e1370)* III;*set-21(ust68)* IV;*ustIS234(daf-16::gfp::3 x flag)* I |
| SHG1896 | *daf-2(e1370)* III;*set-25(n5021)* III;*ustIS234(daf-16::gfp::3 x flag)* I |
| SHG2067 | *daf-2(e1370)* III;*asm-2(tm3746)* X |
| SHG2068 | *daf-2(e1370)* III;*ins-35(ok3297)* V |
| SHG2069 | *daf-2(e1370)* III;*lys-7(ok1384)* V |
| SHG2070 | *daf-2(e1370)* III;*nhr-62(tm1818)* I |
| SHG2071 | *daf-2(e1370)* III;*spp-12(tm2963)* V |
| SHG2072 | *daf-2(e1370)* III;*tts-1(gk105)* X |
| SHG2073 | *daf-2(e1370)* III;*dao-3(ok1678)* X |
| SHG2074 | *daf-2(e1370)* III;*sod-3(tm760)* X |
| SHG2075 | *daf-2(e1370)* III;*Y39G8B.7(ust197)* II |
| SHG2076 | *daf-2(e1370)* III;*F35E8.7(ust198)* V |
| SHG2077 | *daf-2(e1370)* III;*ustIS62(set-13p::set-13::gfp:: 3 x flag)* IV |
