# Supplementary material for: H3K9me1/2 methylation limits the lifespan of daf-2 mutants in C. elegans
Source: eLife. 2022 Sep 20;11:e74812. doi: 10.7554/eLife.74812 (PMC9514849; doi:10.7554/eLife.74812)
Supplement: Supplementary file 3. [file elife-74812-supp3.docx]

**Supplementary file 3.** sgRNA sequences for CRISPR/Cas9-directed gene editing technology.

| Name | Sequence |
| --- | --- |
| *set-13*-sg-1 | AATCCGAATGACCTCCAACATGG |
| *set-13*-sg-2 | CCAGCGTGCCGCTGCTTCCAGAC |
| *set-13*-sg-3 | CGATGTTTGCGTGCTCGGGGAGG |
| *set-21*-sg-1 | CAAATACCACGGGAGGGTTGTGG |
| *set-21*-sg-2 | TGATCGTAGTAATGATCCTTCGG |
| *set-21*-sg-3 | TGACTACGGAAAGACGTACACGG |
| *set-21*-sg-4 | GAACGCTCATTTGAATCTGCTGG |
| *set-21*-sg-5 | TTTCAAACAGCGCGCGCAAAAGG |
| *set-33*-sg-1 | ATGGTGCGTCCATTATTCTGCGG |
| *set-33*-sg-2 | TACGGGCGAGATTGTCGAGCTGG |
| *set-33*-sg-3 | TGAAGCGCATGCACCGCCCGAGG |
| *F35E8.7*-sg-1 | TTGCATTTGTAAAAATAATCTGG |
| *F35E8.7*-sg-2 | TCTACAATTGCACCGATCTGAGG |
| *F35E8.7*-sg-3 | ATGTATTGCCCGAAAACTTGTGG |
| *F35E8.7*-sg-4 | TTTACACGTCTTTCTGCAATTGG |
| *Y39G8B.7*-sg-1 | CAAGTATTCCTACGATGCTCCGG |
| *Y39G8B.7*-sg-2 | ATCAATGCATGGTTTGTCTTGGG |
| *Y39G8B.7*-sg-3 | GACAAAACTTCTTCAGCATGGGG |
| *Y39G8B.7*-sg-4 | ATTGCATTCCTTGCAAGTTCCGG |
