# Supplementary material for: H3K9me1/2 methylation limits the lifespan of daf-2 mutants in C. elegans
Source: eLife. 2022 Sep 20;11:e74812. doi: 10.7554/eLife.74812 (PMC9514849; doi:10.7554/eLife.74812)
Supplement: Supplementary file 4. [file elife-74812-supp4.docx]

**Supplementary file 4.** List of primers used in mRNA qPCR.

| Name | Sequence |
| --- | --- |
| *ama-1* qRT F | CGAACCTGCCGATTGATA |
| *ama-1* qRT R | ACCACGATTGACCAACTC |
| *lys-7*-mRNA-qPCR-F | TGCAGTTTTCGTTCGTGCAT |
| *lys-7*-mRNA-qPCR-R | ACGGGCTGTGGAGTCATATA |
| *spp-12*-mRNA-qPCR-F | TGTGCCCGCAATCTCACTT |
| *spp-12*-mRNA-qPCR-R | AGCTTCCTTGCCATCCTTAA |
| *ins-35*-mRNA-qPCR-F | GATGAGAACGCGTTTGGAAT |
| *ins-35*-mRNA-qPCR-R | CCTTCCATGAGAATCTTTTCATAG |
| *dao-3*-mRNA-qPCR-F | CATTTCGTCAAAGCTGATTGG |
| *dao-3*-mRNA-qPCR-R | CTTCCTTTGCGGCTTCTGT |
| *tts-1*-mRNA-qPCR-F | CGGAGGATTGAGGAAAATTG |
| *tts-1*-mRNA-qPCR-R | ACCTAACTTGCCTGCTTCCA |
| *F35E8.7*-mRNA-qPCR-F | AACACAGGTGCTACGTCCAA |
| *F35E8.7*-mRNA-qPCR-R | GGTCTTTCTTGATCTCCTCCG |
| *nhr-62*-mRNA-qPCR-F | ACACTATGGCGTAAATGCAT |
| *nhr-62*-mRNA-qPCR-R | ACGTTTCGATGTTCTTTTGC |
| *sod-3*-mRNA-qPCR-F | CCGGTTGCGGGAGTTCTC |
| *sod-3*-mRNA-qPCR-R | GGCATGATGCTTTTGATGATG |
| *asm-2*-mRNA-qPCR-F | GCAGCTTTTCTTGTCAACCT |
| *asm-2*-mRNA-qPCR-R | CACAAATCTCTTCCGGTGTG |
| *Y39G8B.7*-mRNA-qPCR-F | CCCAGATTACATCCCCATGC |
| *Y39G8B.7*-mRNA-qPCR-R | ACTAGCACAGTTTGGACTGC |
