# Supplementary material for: H3K9me1/2 methylation limits the lifespan of daf-2 mutants in C. elegans
Source: eLife. 2022 Sep 20;11:e74812. doi: 10.7554/eLife.74812 (PMC9514849; doi:10.7554/eLife.74812)
Supplement: Supplementary file 5. [file elife-74812-supp5.docx]

**Supplementary file 5.** List of primers used in ChIP-qPCR.

| Name | Sequence |
| --- | --- |
| *ama-1* qRT F | CGAACCTGCCGATTGATA |
| ama-1 qRT R | ACCACGATTGACCAACTC |
| *lys-7* promoter ChIP F | TCACACGAAAGTATGTGAAG |
| lys-7 promoter ChIP R | CGGCGTAGTTATCTGAAAAT |
| *spp-12* promoter ChIP F | ACCCTATGACATCATGGAGC |
| *spp-12* promoter ChIP R | GCTACAACCATCCAGGACAT |
| *ins-35* promoter ChIP F | TGACAACTTTGACTTCAAGAAATTG |
| *ins-35* promoter ChIP R | TCAGAAGATCCTAGAGCATTGT |
| *dao-3* promoter ChIP F | GCATATTCAACTTTTTCGACG |
| *dao-3* promoter ChIP R | GGGAAGAGACTAGTGCAGAA |
| *tts-1* promoter ChIP F | GGTTCATGTTTACCAGTCAC |
| *tts-1* promoter ChIP R | GAACACAGCCCTATCAAAGG |
| *F35E8.7* promoter ChIP F | GCTCTTTGGCCTACTTTTAG |
| *F35E8.7* promoter ChIP R | CAAATGCAAGAGGTAGTGAT |
| *nhr-62* promoter ChIP F | AGGCTACAAAATCCTCTTGC |
| *nhr-62* promoter ChIP R | TGATCGGATATGAATGGGCG |
| *sod-3* promoter ChIP F | GGGTTGTTTACGCGTTTTCA |
| *sod-3* promoter ChIP R | GAAGATTTGACAAACGGTCAC |
| *asm-2* promoter ChIP F | CCATCGATAAATATGAAATGTACAGG |
| *asm-2* promoter ChIP R | ATCAACAGTTCTAGCCGACC |
| *Y39G8B.7* promoter ChIP F | ACTCGGAAATGCTGATGTAC |
| *Y39G8B.7* promoter ChIP R | GATTGAAGATGACTGCTCGC |
