# Supplementary material for: H3K9me1/2 methylation limits the lifespan of daf-2 mutants in C. elegans
Source: eLife. 2022 Sep 20;11:e74812. doi: 10.7554/eLife.74812 (PMC9514849; doi:10.7554/eLife.74812)
Supplement: Supplementary file 6. [file elife-74812-supp6.docx]

**Supplementary file 6** Published ChIP-seq datasets used in the study.

| TF | Stage | Website |
| --- | --- | --- |
| DAF-16 | Mix Culture | https://sra-downloadb.be-md.ncbi.nlm.nih.gov/sos1/sra-pub-run-5/SRR1977494/SRR1977494.1 |
| DAF-16 input | Mix Culture | https://sra-downloadb.be-md.ncbi.nlm.nih.gov/sos1/sra-pub-run-5/SRR1977495/SRR1977495.1 |
| CBP-1 | Emb | https://sra-downloadb.be-md.ncbi.nlm.nih.gov/sos1/sra-pub-run-1/SRR8201394/SRR8201394.1 |
| PHA-4 | Emb | https://sra-downloadb.be-md.ncbi.nlm.nih.gov/sos1/sra-pub-run-1/SRR8201407/SRR8201407.1 |
| HSF-1 | YA | https://sra-downloadb.be-md.ncbi.nlm.nih.gov/sos1/sra-pub-run-1/SRR3535769/SRR3535769.1 |
| NHR-80 | YA | https://www.encodeproject.org/files/ENCFF728BOT/download/ENCFF728BOT.fastq.gz |
| PQM-1 | YA | https://sra-downloadb.be-md.ncbi.nlm.nih.gov/sos1/sra-pub-run-2/SRR8255717/SRR8255717.1 |
| ELT-3 | L3 | https://www.encodeproject.org/files/ENCFF255JOZ/download/ENCFF255JOZ.fastq.gz |
