# Supplementary material for: H3K9me1/2 methylation limits the lifespan of daf-2 mutants in C. elegans
Source: eLife. 2022 Sep 20;11:e74812. doi: 10.7554/eLife.74812 (PMC9514849; doi:10.7554/eLife.74812)
Supplement: Source code 1. [file elife-74812-code1.zip › 18-10-2021-RA-eLife-74812/Source Code File 1.docx]

# Alignment and peak calling

for i in *.fastq.gz; do echo $i;

bowtie2 -p 24 –x bt2_wbcel235 -q $i -S $i.sam;

done

ls *.sam|cut -d"." -f 1 |while read id ; do

samtools view -Sb -q 10 $id.sam > $id.bam

done

ls *.bam|cut -d"." -f 1 |while read id ; do

samtools sort $id.bam -o $id.sorted.bam

done

ls *sorted.bam|cut -d"." -f 1,2 |while read id ; do

samtools index $id.bam

done

ls *.bam|cut -d"." -f 1 |while read id ; do

macs2 callpeak -t $id.bam -c input.$id.bam -g ce -B -f BAM -n $id.sorted.peaks -q 0.01 -m 4 50;

done

#Data visualization

ls *sorted.bam|cut -d"." -f 1,2 |while read id ; do

bamCoverage -b $id.bam -o $id.bw --binSize 20 --normalizeUsing BPM --smoothLength 60 --extendReads 150 --centerReads -p 6 2> $id.bamCoverage.log;

done

ls *.bw|cut -d"." -f 1 |while read id ; do

computeMatrix scale-regions -S $id.bw -R $id.bed -b 500 -a 500 --skipZeros -out $id.scale.gz --outFileSortedRegions $id.matrix.scale.bed;

done

ls *.gz|cut -d"." -f 1 |while read id ; do

plotProfile -m $id.scale.gz –out $id.pdf --yMax 8
